# Latent Classes of Adolescent Trauma Exposure, Posttraumatic Stress Disorder Symptoms, and Substance Use Predict Clinical Diagnoses at 12-Month Follow-Up

**DOI:** 10.1177/24705470251350144

**Published:** 2025-06-20

**Authors:** John Leri, Josh M. Cisler, Shaunna L. Clark, Cody G. Dodd, Saman Siddiqui, Leslie Taylor, Alexa Ayala, Sunita Stewart, Robyn Richmond, Jeffrey D. Shahidullah, Justin F. Rousseau, John M. Hettema, D. Jeffrey Newport, Karen D. Wagner, Charles B. Nemeroff

**Affiliations:** 1 Department of Psychiatry and Behavioral Sciences, 377659 The University of Texas at Austin Dell Medical School, Austin, TX, USA; 2 Department of Psychiatry & Behavioral Sciences, 14736 Texas A&M University, College Station, TX, USA; 3 Department of Psychiatry and Behavioral Sciences, 6177 The University of Texas Medical Branch, Galveston, TX, USA; 4 23240 JPS Health Network, Fort Worth, TX, USA; 5 Faillace Department of Psychiatry and Behavioral Sciences, 12340 The University of Texas Health Science Center at Houston, Houston, TX, USA; 6 Department of Psychiatry and Behavioral Sciences, 12346 The University of Texas at San Antonio, San Antonio, TX, USA; 7 Department of Psychiatry, 12334 The University of Texas Southwestern Medical Center, Dallas, TX, USA; 8 Department of Surgery, 21976 Texas Tech University Health Sciences Center, Lubbock, TX, USA; 9 Department of Neurology, 12334 The University of Texas Southwestern Medical Center, Dallas, TX, USA; 10 21976 The University of Texas Southwestern Medical Center, The Peter O’Donnell Jr. Brain Institute, Dallas, TX, USA; 11 Department of Women's Health, 377659 The University of Texas at Austin Dell Medical School, Austin, TX, USA

**Keywords:** trauma, posttraumatic stress disorder, substance use disorder, adolescence, youth, polysubstance use, interpersonal victimization, latent class analysis, latent profile analysis, development

## Abstract

**Background:**

Trauma exposure, posttraumatic stress disorder (PTSD), and substance use commonly co-occur among youth. Identifying specific subgroups of youth based on unique constellations across these domains may provide a novel way to identify and target youth at prospective risk for specific types of negative clinical outcomes.

**Methods:**

Trauma exposed youth completed structured clinical assessments as part of a longitudinal study (*N* = 1826; ages 13-21). Latent class analyses identified distinct subgroups of youth based on lifetime trauma histories and current PTSD symptom and substance use inventories collected at the baseline study visit. Logistic regression analyses determined if the latent classes were associated with elevated risk for PTSD or substance use disorder (SUD) diagnoses as the 12-month follow-up study visit (*n* = 1029). Logistic regression models controlled for baseline clinical characteristics and demographic factors in a stepwise fashion to elucidate if latent classes carried conferred risk beyond established risk factors. Sensitivity analyses included latent profile analyses and predictive modeling with an alternative number of latent classes.

**Results:**

Four latent classes were identified which differentiated participants based on the type of trauma exposure, the number of PTSD symptoms endorsed, and the propensity to be engaged in polysubstance use. Latent classes which were characterized by exposure to interpersonal violence at the baseline study visit had an elevated risk of PTSD 12 months later, relative to the latent class which was principally exposed to incidental trauma (odds ratios ranged from 4.11-5.88). Likewise, a distinct latent class which was characterized by poly-substance use at the baseline study visit had an elevated risk of SUD diagnoses at the 12-month follow-up (odds ratio = 2.48). The findings were robust to sensitivity analyses.

**Conclusion:**

These results highlight nuanced patterns of co-occurrences between trauma exposure, PTSD symptomatology, and substance use that differentiate unique sub-groups of youth at varying degrees of risk for negative clinical outcomes one year later. Evaluating the co-expression of trauma and psychopathology inventories, as opposed to only assessing the summative epidemiological indices of these constructs, may help identify adolescents who are most at risk for sustaining deleterious health outcomes.

## Introduction

Early life trauma exposure is an antecedent to both posttraumatic stress disorder (PTSD) and substance use disorder (SUD), two common diagnoses that are often comorbid. Nevertheless, many individuals exposed to trauma will not exhibit symptoms of PTSD^1^ or engage in substance use.^2^ Likewise, of those who display symptoms of PTSD or engage in substance use, there is heterogeneity in terms of the number or severity of PTSD symptoms experienced^3,4^ and the types of substance use behavior that is engaged in.^5,6^ Understanding how specific types of traumas, PTSD symptoms, and substance use co-occur may highlight the most deleterious groupings and inform interventions that target specific typologies; a strategy which may be particularly impactful during adolescence, a developmental stage when many psychiatric conditions first emerge.

Mixture models have been used to identify latent subgroups, otherwise referred to as classes or profiles depending on the data structure, of adolescents based on their multivariate response patterns to trauma, PTSD, or substance use inventories when considered in isolation (eg, latent classes of trauma exposure only). Adolescent trauma inventories typically break down into no- or incidental-trauma exposures, high levels of poly-trauma exposures, and groups characterized by specific types of trauma exposures (eg, sexual assault or caregiver abuse).^7–9^ Likewise, substance use behaviors differentiate adolescents into no-substance use groups, poly-substance users, and mono-substance users defined by a primary substance (eg, alcohol only),^10–12^ whereas PTSD inventories can delineate adolescents by how widespread symptom expression is and expression of distinct symptom clusters.^13,14^ These findings suggest that meaningful subgroups can be formed from adolescent reports of trauma, PTSD, and substance use, which may have clinical utility.

Latent subgroups derived from trauma, PTSD, or substance use inventories have been used to predict scores on related conceptual inventories. For example, adolescents that fit a poly-trauma profile have been identified as more likely to exhibit concurrent problem drinking behaviors than adolescents exposed to incidental traumas.^15^ These findings are broadly concordant with results produced by other analytical techniques (eg, structural equation modeling), because, trauma, PTSD symptoms, and substance use tend to covary.^16^ Nevertheless, executing mixture models on a single concept (eg, PTSD symptoms) presupposes that all the concepts of interest (eg, PTSD symptoms, substance use, and trauma exposure) do not co-occur systematically. Indeed, mixture models which have been applied concurrently across PTSD and substance use inventories among adults have identified groups which exhibit high or low PTSD symptoms and corresponding levels of substance use, in addition to groups with more moderate PTSD symptoms that are distinguished by variation in specific types of substance use.^17–19^ These results indicate that latent subgroups may be more informative when derived from multiple interrelated conceptual inventories.

The current study aimed to use a latent class analysis (LCA) to identify patterns of heterogeneity among trauma, PTSD symptoms, and substance use among a cohort of trauma exposed youth (adolescents and emerging adults). This aim extends extant research by modeling all three conceptual inventories within the LCA framework, as opposed to performing LCA on a specific subset of clinical constructs. The use of LCA to identify subgroups of youth also furthers previous work with this cohort which has reported that on average, higher levels of trauma exposure predict higher levels of PTSD symptom severity and substance use behaviors,^10,16,20^ but have not necessarily identified the specific subgroups of youth for whom trauma antecedes PTSD symptomology and problematic substance use. Further, we aimed to determine if latent classes derived from conjoined trauma, PTSD symptoms, and substance use indicators indexed at the baseline study visit may have clinical utility, such that the latent classes were used to predict longitudinal PTSD and SUD diagnoses at the 12-month follow-up study visit. These aims collectively sought to identify youth at risk for deleterious trauma-related psychiatric sequalae.

We hypothesized that at least three latent classes would be identified. Specifically, we expected to observe a class characterized by a broad range of trauma exposures with high levels of PTSD symptoms and polysubstance use, a class with low levels of trauma exposure and relatively low levels of PTSD symptoms and substance use, and a class with moderate levels of trauma exposure with moderate levels PTSD symptoms and substance use. Additional classes of youth were expected to be differentiated based on exposure to specific types of traumas (eg, sexual assault), expression of distinct subclusters of PTSD symptoms (eg, hyperreactive vs not), or preferences for specific types of substances. We further hypothesized that specific latent classes identified from data collected at the baseline study visit would be at risk for longitudinal PTSD and SUD diagnoses. Specifically, we expected that the latent classes with the most severe PTSD symptoms or the highest trauma burden would be at elevated risk for PTSD diagnoses at the 12-month follow-up study visit. Latent classes with higher probabilities of polysubstance use were hypothesized to be at higher risk for SUD at the 12-month follow-up visit.

## Methods

### Procedures

This study used data collected by the Texas Childhood Trauma Research Network,^10,16,20–23^ a research initiative of the Texas Child Mental Health Care Consortium (TCMHCC). Youth (ages 8 to 20) and their caregivers were recruited to participate by twelve academic medical centers located across Texas. All study procedures were approved by the Institutional Review Board (IRB) at the University of Texas Southwestern and instantiated via IRB reliance agreements with the other eleven institutions. Interested youth and caregivers provided informed consent or assent as applicable and were then screened by study personnel for a qualifying trauma exposure and comprehension of English or Spanish. Exclusion criteria included psychotic symptoms, developmental disorders, and severe intellectual disability. Eligible youth were enrolled in the study and scheduled for a baseline study visit in addition to 1-, 6-, 12-, 18-, and 24-month follow-up study visits.

Self-report assessments and structured interviews were administered by trained study staff to index youth's experiences of trauma, PTSD symptoms, and substance use.^22^ Trauma exposure was measured at all study visits except for the 1-month follow-up. PTSD, substance use, use of psychiatric medication, psychotherapy involvement, and psychiatric hospitalizations were assessed at all study visits. Self-reported demographic and family medical histories were collected at the baseline study visit. The current study leverages data collected at the baseline and 12-month follow-up study visits.

### Measures

#### Trauma Exposure

Trained interviewers administered the Traumatic Events Screening Inventory for Children (TESI-C);^24^ to measure youth's lifetime exposure to 20 types of trauma experiences (see Table S1). Youth's descriptions of each exposure were used to determine if the exposure qualified as a DSM-5 PTSD criterion A exposure. Item responses were coded as one if the exposure met criterion A and zero if youth were not exposed or the exposure did not meet criterion A.^20^

#### Posttraumatic Stress Disorder

Trained interviewers administered the Clinician-Administered PTSD Scale for DSM-5 Child/Adolescent Version (CAPS-CA-5) to youth.^25^ Interviewers conducted a structured interview that produced severity scores for the 20 items constituting the re-experiencing, avoidance, negative alterations in cognition and mood, and arousal and reactivity symptom clusters of DSM-5 PTSD. Item severity scores, which could range from 0 to 4, were coded as binary indictors for the latent class analysis. Item severity scores of 0 (absent) or 1 (mild/subthreshold) were denoted as zero and item severity scores of 2 (moderate/threshold), 3 (severe/markedly elevated), and 4 (extreme/incapacitating) were denoted as one, consistent with the scoring instructions for indicating the presence of a symptom which causes either significant distress or functional impairment.^25^ PTSD diagnostic status was determined according to the publisher's directions.^25^

#### Substance Use

Four items from the CRAFFT 2.1 + N (Car, Relax, Alone, Forget, Friends, Trouble: Version 2.1 + Nicotine)^26^ were used to assess substance use. Youth self-reported the number of days on which they consumed alcohol, cannabis products, nicotine, or other drugs during the previous 12-months. Responses were coded as binary indicators such that zero and one, respectively, indicated a substance had not or had been used during the previous 12-months. Diagnoses for alcohol use disorder and substance use disorder were derived from the Mini-International Neuropsychiatric Interview for Children and Adolescents (MINI-KID),^27^ a structured diagnostic interview. A binary substance use disorder indicator variable was created from the MINI-KID diagnoses for which 0 indicated the absence of any type of substance use disorder and 1 indicated the presence of either an alcohol use disorder or non-alcohol substance use disorder.

### Data Analysis

Statistical analyses were executed using R statistical software, version 4.3.1. The full sample consisted of 2496 youth (enrollment October 2020 through March 2024). Because there were a limited number of youth who endorsed substance use before age 13, the analytical sample was restricted to youth ages 13 through 20 (*n* = 1826). Latent class analyses were executed using the ‘poLCA’ package.^28^ A total of 44 binary (0/1) indicators from the baseline study visit (20 trauma indicators, 20 PTSD indicators, and 4 substance use indicators) were used as input for the LCA. Item level data were used, as opposed to composite scores of inventory subdimensions, to maximize the parameter space used to differentiate latent classes. A total of 1291 participants had complete data for LCA model enumeration. LCA solutions were enumerated for 1 through 10 classes via maximum likelihood estimation implemented with an expectation-maximization algorithm. Model solutions for each number of classes were estimated multiple times (*n* = 10) to ensure that the algorithm converged on a global maximum of the log-likelihood function. Starting parameters were selected at random for each model estimation. Akaike information criterion (AIC), Bayesian information criterion (BIC), and the bootstrapped likelihood ratio test (BLRT), were used to compare the relative fit of the LCA solutions. Entropy was used to assess the differentiation between LCA classes for each model solution. Additional considerations for selecting an optimal number of classes included the proportion of youth assigned to each class and the conceptual interpretability of each class.

After a latent class solution was selected, participants were assigned to the latent classes with the highest posterior probability. Latent class assignments were then used to predict SUD and PTSD diagnoses at the 12-month follow-up study visit.

Prior to the predictive analyses, the ‘mice’ package^29^ was used to perform multiple imputation for missing covariates from the baseline study visit and selected data elements at the follow-up study visits. Specifically, follow-up data were imputed if participants were lost to follow-up or if the follow-up study visit was completed outside of the accepted range (+- 30 days of the scheduled study visit). Missing data were not imputed for participants who had not matriculated to the follow-up study visits. Logistic regression and predictive mean matching, respectively, were used to impute categorical and continuous variables. After missing data were imputed there were 1029 participants with clinical diagnostic data at the 12-month study visit.

Nested logistic regressions were used to determine if latent class membership predicted 12-month PTSD and SUD diagnoses. Each nested regression added a block of variables to the models which predicted 12-month diagnostic status. The null model included only an intercept. Step-1 added the latent classes to the model. Step-2 added lifetime trauma exposure, PTSD diagnoses, and SUD diagnoses from the baseline study visit. Step-3 added trauma exposure, psychotherapy involvement, psychotropic medication use, and psychiatric hospitalizations which occurred between the baseline study visit and the 12-month study visit. Step-4 added demographic variables including age, sex, race, ethnicity, household income, and family history of psychiatric and substance use disorders. Models were fitted on all imputed datasets (*n* = 100) and parameter estimates were pooled according to Rubin's rules.^30^ The relative fit of the nested models was compared using the multivariate Wald test.

## Results

### Descriptives

See Table 3 for descriptive statistics of the analytic sample. In brief, the analytic sample was composed predominately of female (63%) youth, aged 13 to 20 (*M* = 16.83, *SD* = 2.26), from households across the socioeconomic spectrum (<$25,000 = 25%, $25,000 - $99,999 = 50%, > $99,999 = 25%). A large proportion of youth identified as White (65%) but there were also substantial proportions of both Black (15%) and Multiracial youth (12%); nearly half the sample identified as Hispanic (47%). There was a large range in the types of trauma exposures (up to 18 types of trauma exposures endorsed), variability in PTSD symptom severity (*M* = 6.72, *SD* = 5.22), and endorsement of substance use (38% of youth endorsed substance use in the previous 12-months at the baseline study visit). The prevalence of clinical PTSD diagnoses at the baseline and 12-month follow-up study visits was 28% and 18%; for clinical substance use disorder diagnoses, the prevalence was 14% and 11%, respectively.

### Primary Analyses

**Figure 1.**
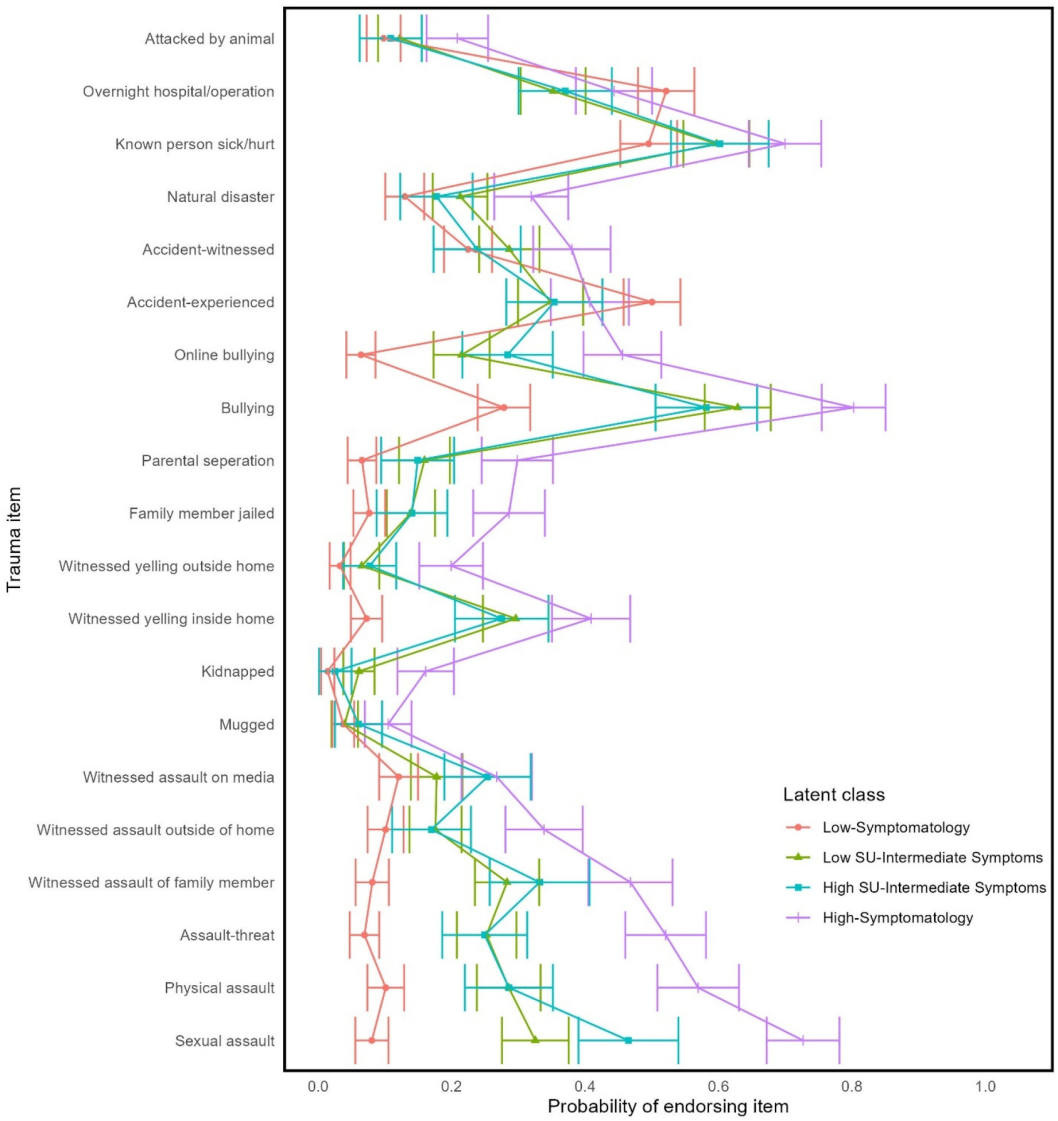
Probability of endorsing trauma items by latent class membership.

Note. Figure shows the posterior probability of endorsing each trauma item and the 95% confidence interval for each class. SU = substance use.

**Figure 2.**
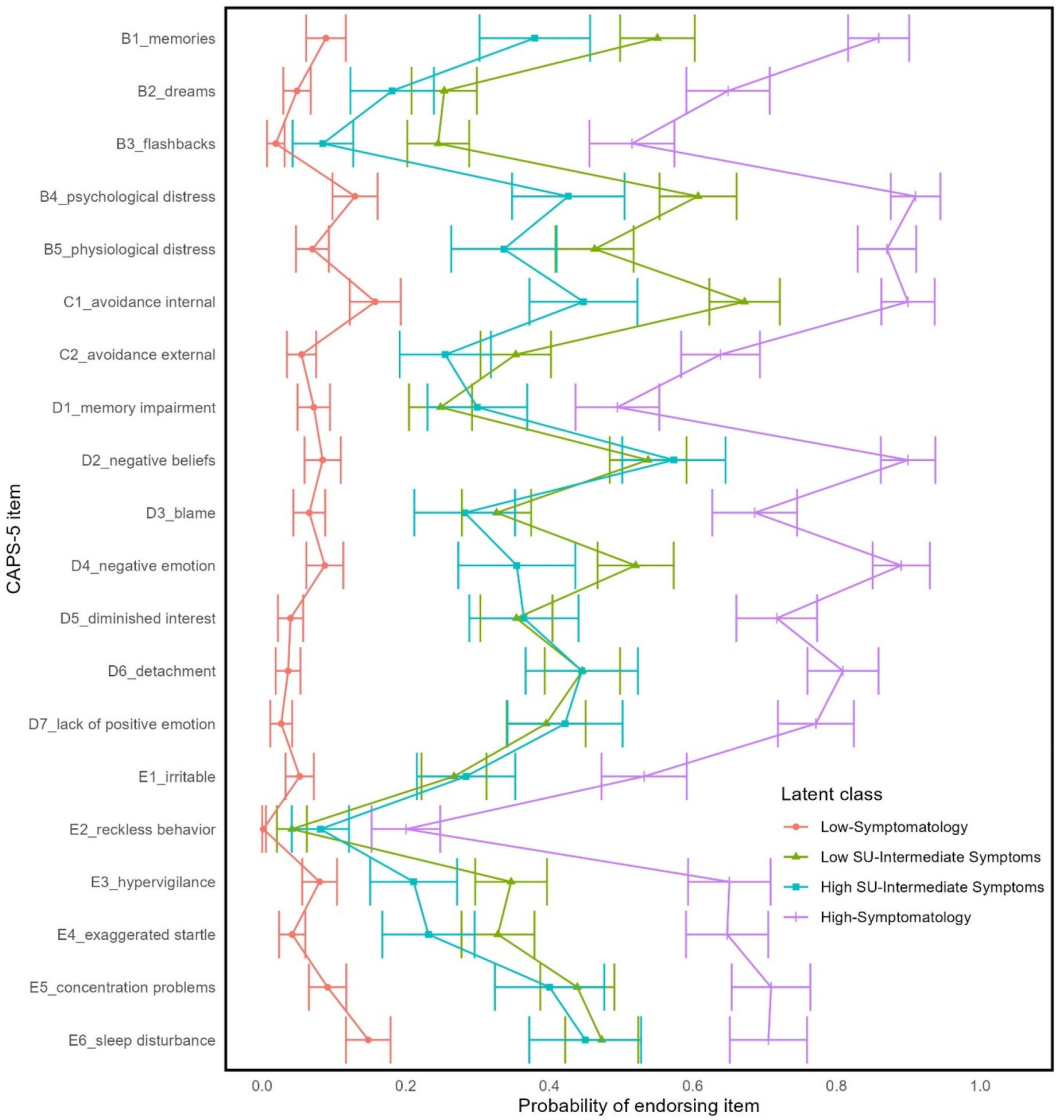
Probability of endorsing a posttraumatic stress disorder symptom by latent class membership.

Note. Figure shows the posterior probability of endorsing each CAPS-CA-5 and the 95% confidence interval for each class. SU = substance use.

**Figure 3.**
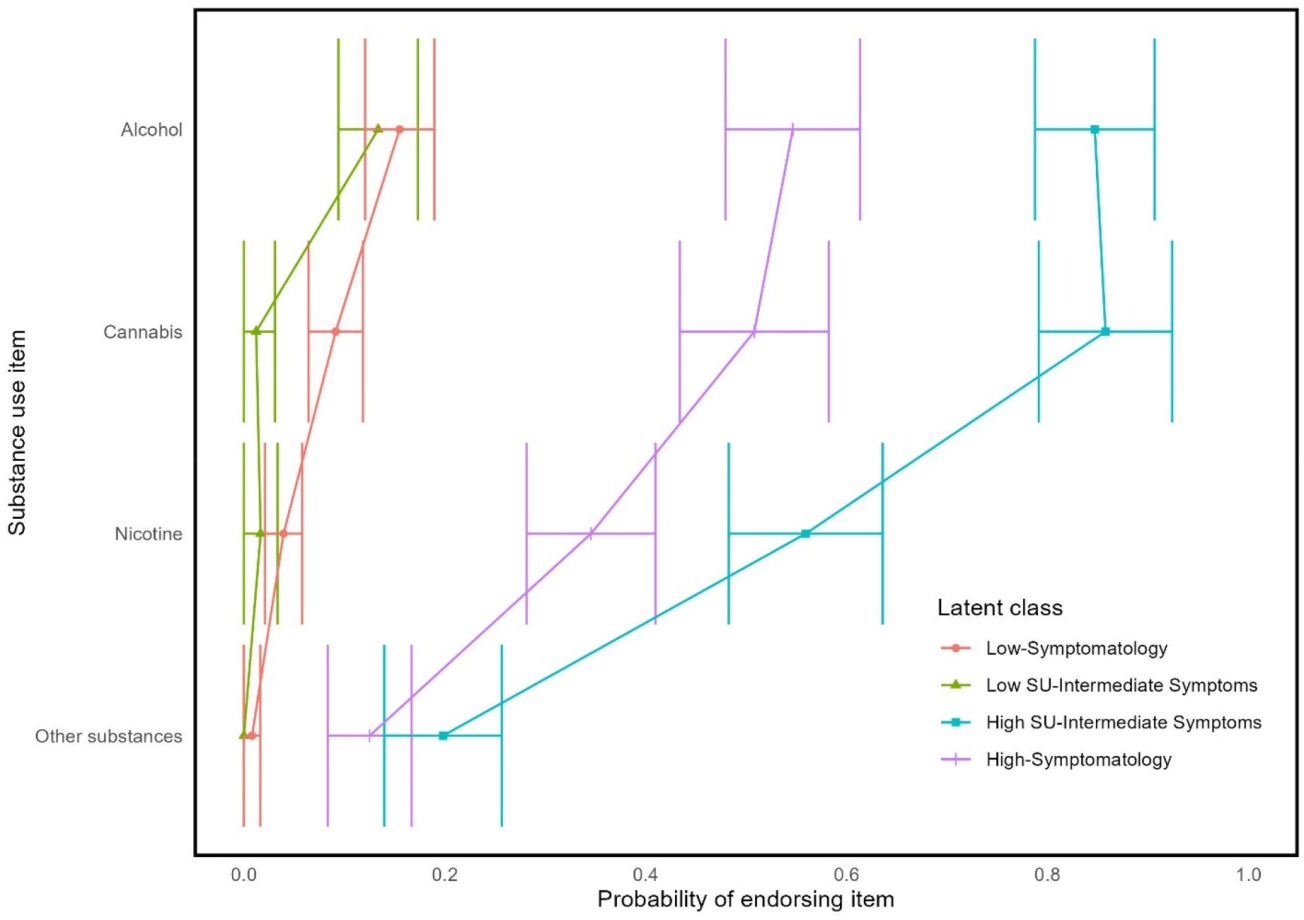
Probability of endorsing substance use items by latent class membership.

Note. Figure shows the posterior probability of endorsing each substance use item and the 95% confidence interval for each class. SU = substance use.

Model solutions with up to 10 classes were fitted. The four-class solution was deemed optimal based on the relative model fit indices (Table 1). Specifically, as indicated by the BLRT *p*-value, there was a significant improvement in model fit when a fourth class was added to the model (BLRT *p* = .009), whereas models with more than four classes did not significantly improve model fit (all BLRT *p* ≥ .188). Consistent with the four-class solution being optimal, models beyond the four-class solution yielded relatively smaller improvements to AIC and BIC statistics. The four-class solution was also favored for its sizable proportions of youth in each latent class (all classes >= 15% of the sample; Table 2) and conceptually coherent classes. Table 3 shows descriptive statistics for each latent class. The probabilities of each latent class endorsing individual trauma, PTSD, and substance use indicators are shown in Figures 1–3, respectively.

**Table 1.** Model fit Statistics per Each n-Class Model Solution.

N-classes	Loglikelihood	AIC	BIC	Entropy	BLRT *p*
1	−42669.84	85471.67	85670.11	−	−
2	−38429.70	77126.40	77527.78	0.89	.009
3	−37673.70	75749.40	76353.72	0.85	.009
4	−37314.35	75165.69	75972.97	0.84	.009
5	−37112.47	74896.93	75907.15	0.82	.188
6	−36874.92	74556.83	75769.99	0.82	.168
7	−36695.22	74332.44	75748.55	0.82	.238
8	−36554.37	74185.74	75804.79	0.81	.208
9	−36451.77	74115.53	75937.53	0.81	.396
10	−36354.60	74056.20	76081.14	0.82	.416

Note. AIC = Akaike information criterion, BIC = Bayesian information criterion, BLRT = bootstrapped likelihood ratio test.

**Table 2.** Proportion of Participants in Each Class per Each n-Class Model Solution.

N-classes	Class 1	Class 2	Class 3	Class 4	Class 5	Class 6	Class 7	Class 8	Class 9	Class 10
1	100%	−	−	−	−	−	−	−	−	−
2	49%	51%	−	−	−	−	−	−	−	−
3	24%	36%	40%	−	−	−	−	−	−	−
4	28%	21%	36%	15%	−	−	−	−	−	−
5	12%	23%	17%	35%	13%	−	−	−	−	−
6	16%	14%	30%	16%	12%	12%	−	−	−	−
7	19%	12%	10%	12%	14%	25%	9%	−	−	−
8	8%	14%	23%	14%	9%	10%	9%	13%	−	−
9	9%	9%	9%	7%	13%	16%	8%	22%	8%	−
10	8%	11%	6%	7%	5%	25%	7%	9%	8%	14%

Note. Class designations are arbitrary across model solutions, which means that the phenotype which describes Class 1 for the 2-class solution is not necessarily the same phenotype which is described by Class 1 in the 3-class solution.

**Table 3.** Demographic and Summary Variables Presented for the Analytic Sample and Each Latent Class Derived from the Four-Class Solution.

	Whole Sample	Low-Symptomology	Low Substance Use with Intermediate Symptoms	High Substance Use-Intermediate Symptoms	High-Symptomatology
N or proportion of N	1826	36%	28%	15%	21%
Age	16.83 (2.26)	16.00 (2.24)	16.97 (2.20)	17.74 (1.93)	17.47 (2.11)
Female	63%	41%	72%	66%	85%
Race					
White	65%	62%	66%	63%	66%
Black	15%	19%	13%	14%	13%
Other	8%	8%	8%	12%	6%
Multiracial	12%	11%	13%	11%	15%
Hispanic	47%	44%	53%	46%	47%
Household income					
< $25,000	25%	19%	29%	27%	27%
$25,000 - $99,999	50%	49%	49%	52%	52%
> $99,999	25%	32%	22%	21%	21%
Criterion A lifetime exposure	4 (0-18)	3 (0-12)	5 (1-13)	5 (0-15)	8 (1-18)
Unintentional	2 (0-7)	2 (0-7)	2 (0-7)	2 (0-6)	3 (0-7)
Intentional interpersonal	1(0-5)	0 (0-3)	1 (0-4)	1 (0-4)	2 (0-5)
Bullying	1 (0-2)	0 (0-2)	1 (0-2)	1 (0-2)	1 (0-2)
Intentional in-home	0 (0-3)	0 (0-3)	0 (0-3)	0 (0-3)	1 (0-3)
Intentional out-of-home	0 (0-3)	0 (0-3)	0 (0-3)	0 (0-3)	1 (0-3)
PTSD symptom severity	17.57 (13.99)	4.61 (4.04)	20.04 (7.74)	17.29 (7.95)	36.48 (11.04)
Cluster B	4.47 (4.15)	1.21 (1.64)	5.25 (3.12)	3.60 (2.73)	9.53 (3.71)
Cluster C	2.13 (1.99)	0.70 (1.04)	2.63 (1.78)	1.91 (1.64)	4.04 (1.80)
Cluster D	6.56 (5.87)	1.44 (1.75)	7.26 (4.13)	7.40 (4.29)	13.78 (4.93)
Cluster E	4.40 (4.12)	1.26 (1.75)	4.89 (3.16)	4.37 (3.24)	9.13 (3.85)
Substance use					
No substance use	62%	81%	86%	6%	36%
Mono substance use	15%	13%	14%	15%	20%
Poly substance use	23%	6%	0%	79%	43%
Alcohol	33%	15%	13%	86%	55%
Cannabis	27%	9%	1%	88%	51%
Nicotine	17%	4%	1%	59%	34%
Other	6%	1%	0%	20%	13%
PTSD diagnosis					
Baseline study visit	28%	0%	29%	16%	81%
12-month study visit	18%	1%	18%	25%	41%
SUD diagnosis					
Baseline study visit	14%	5%	3%	50%	34%
12-month study visit	11%	5%	5%	47%	26%
Family history					
Substance use disorder	24%	14%	21%	34%	36%
Psychiatric disorder	53%	38%	54%	60%	73%

Notes. Mean and standard deviation shown for age, total PTSD symptom severity, and PTSD symptom cluster severity. Median, minimum, and maximum values are shown for criterion A lifetime trauma exposures and subcategories of trauma exposure. Subcategories of trauma exposure are derived from Aksan and colleagues.^20^

Proportions are shown for categorical variables.

The largest latent class (36% of sample), the ‘Low-Symptomatology’ class, had a low probability of endorsing most traumas, PTSD symptoms, and substance use indicators. The Low Symptomatology class was relatively unlikely to have experienced interpersonal trauma and most likely to have been in an accident or have had an overnight hospitalization or surgery. If the Low-Symptomatology class engaged in substance use, it was most likely to be alcohol or cannabis.

The ‘High-Symptomatology’ class (21% of sample) had a moderate to high probability of having been exposed to any given trauma and were most likely to have experienced any of the interpersonal traumas. This class also had the highest probability of endorsing any of the PTSD symptoms and had high probabilities of having consumed alcohol, cannabis, nicotine, and other substances.

There were two latent classes that were intermediate to the Low-Symptomatology and High-Symptomatology classes. The two classes were differentiated from each other based on patterns of substance use. Specifically, the ‘High Substance Use with Intermediate Symptom’ class (15% of sample) had high probabilities of endorsing multiple types of substance use, whereas the ‘Low Substance Use with Intermediate Symptom’ class (28% of sample) had low probabilities of endorsing any substance use. Both had moderate probabilities of endorsing any given trauma exposure, which includes both accidental and interpersonal trauma. Likewise, both classes had moderate probabilities of endorsing any given PTSD symptom.

Logistic regressions were fitted that predicted clinical PTSD diagnoses at the 12-month follow-up visit. Step-1 showed that the latent classes significantly improved model fit compared to the intercept only model (*F_(3, 1025)_* = 19.29, *p* < .001). Specifically, relative to the Low-Symptomatology class, the other latent classes were significantly more likely to have a PTSD diagnosis at the 12-month follow-up visit (odds ratios ranged from 8.93-25.02). Step-2 indicated that lifetime trauma exposure, PTSD diagnoses, and SUD diagnoses from the baseline visit improved model fit (*F_(3, 1022)_* = 6.10, *p* = .001); as did the inclusion of recurrent trauma and psychiatric therapy involvement during the inter-visit interval within Step-3 (*F_(4, 1018)_* = 8.92, *p* < .001). The addition of demographic covariates in Step-4 did not significantly improve model fit when predicting 12-month PTSD diagnoses (*F_(10, 1008)_* = 1.08, *p* = .374). Although the inclusion of covariates attenuated the association between the latent classes and 12-month PTSD diagnoses (odds ratios ranged from 4.17-5.88 in the fully adjusted model), the Intermediate Symptom and High-Symptomatology latent classes were all significantly more likely to have a PTSD diagnosis at the 12-month study visit relative to the Low-Symptomatology class. In the fully adjusted model, the odds ratios of 12-month PTSD diagnoses were not significantly different between the Intermediate Symptom and High-Symptomatology latent classes (all *p* >= .277). See Table 4 for the parameter estimates of each nested regression.

**Table 4.** Odds Ratios and 95% Confidence Intervals for the Logistics Regressions Which Predict Posttraumatic Stress Disorder Diagnoses at the 12-Month Study Visit Based on the Latent Classes Derived from the Baseline Study Visit.

.	Model 1			Model 2			Model 3			Model 4		
	*OR*	*95% CI LL*	*95% CI UL*	*OR*	*95% CI LL*	*95% CI UL*	*OR*	*95% CI LL*	*95% CI UL*	*OR*	*95% CI LL*	*95% CI UL*
Latent classes												
Low Symptomatology	Ref	Ref	Ref	Ref	Ref	Ref	Ref	Ref	Ref	Ref	Ref	Ref
Low Substance Use-Intermediate Symptoms	**8.93**	**3.37**	**23.65**	**6.17**	**2.24**	**17.02**	**4.00**	**1.44**	**11.09**	**4.17**	**1.48**	**11.78**
High Substance Use-Intermediate Symptoms	**10.35**	**3.73**	**28.75**	**7.02**	**2.39**	**20.61**	**5.15**	**1.73**	**15.29**	**5.66**	**1.82**	**17.57**
High Symptomatology	**25.02**	**9.60**	**65.25**	**9.61**	**3.08**	**29.98**	**5.88**	**1.85**	**18.65**	**5.88**	**1.80**	**19.24**
Baseline PTSD diagnosis				**2.79**	**1.68**	**4.62**	**2.83**	**1.66**	**4.80**	**3.01**	**1.74**	**5.20**
Baseline SUD diagnosis				1.46	0.88	2.44	1.16	0.67	2.02	1.06	0.59	1.89
Criterion A lifetime trauma				0.99	0.92	1.07	0.95	0.87	1.03	0.95	0.87	1.04
Criterion A trauma exposure between study visits							**1.26**	**1.15**	**1.39**	**1.30**	**1.17**	**1.44**
Psychotropic medication between study visits							**1.69**	**1.06**	**2.70**	**1.83**	**1.12**	**3.00**
Psychotherapy between study visits							1.80	0.89	3.64	1.80	0.86	3.76
Psychiatric hospitalization between study visits							1.21	0.73	1.99	1.16	0.69	1.94
Age										0.99	0.88	1.11
Sex												
Female										Ref	Ref	Ref
Male										1.16	0.70	1.94
Race												
White										Ref	Ref	Ref
Black										0.59	0.26	1.34
Other minority										0.86	0.37	2.02
Multiracial										1.55	0.82	2.93
Hispanic										1.39	0.85	2.28
Household income												
< $25,000										Ref	Ref	Ref
$25,000 - $99,999										0.80	0.44	1.45
> $99,999										1.31	0.65	2.63
Family history												
Psychiatric disorders										0.97	0.57	1.62
Substance use disorders										0.96	0.57	1.62

Model comparison	*F*	*df1*	*df2*	*p*								
Model 1 v 0	**19.29**	**3**	**1025**	**< .001**								
Model 2 v 1	**6.10**	**3**	**1022**	.**001**								
Model 3 v 2	**8.92**	**4**	**1018**	**< .001**								
Model 4 v 3	1.08	10	1008	.374								

Logistic regressions were fitted that predicted clinical SUD diagnoses at the 12-month follow-up visit. Step-1 showed that the latent classes significantly improved model fit compared to the intercept only model (*F_(3, 1025)_* = 28.56, *p* < .001). Relative to the Low-Symptomatology class, the High Substance Use with Intermediate Symptom (odds ratio = 11.41) and High-Symptomatology (odds ratio = 5.41) latent classes were significantly more likely to have a SUD diagnosis at the 12-month follow-up visit. Step-2 indicated that lifetime trauma exposure, PTSD diagnoses, and SUD diagnoses from the baseline visit improved model fit (*F_(3, 1022)_* = 27.88, *p* < .001) as did the inclusion of recurrent trauma and psychiatric therapy involvement during the inter-visit interval within Step-3 (*F_(4, 1018)_* = 2.99, *p* = .018). The addition of demographic covariates in Step-4 did not significantly improve model fit when predicting 12-month SUD diagnoses (*F_(10, 1008)_* = 1.38, *p* = .186). The inclusion of covariates attenuated the association between the High Substance Use with Intermediate Symptom class and 12-month SUD diagnosis to marginal significance (odds ratio = 2.48) and nullified the association between the High-Symptomatology class and 12-month SUD diagnoses (odds ratio = 0.84). In the fully adjusted model, the odds ratio of the High Substance Use with Intermediate Symptoms class was larger than the Low Substance Use with Intermediate Symptoms class (*p* < .001) and the Low-Symptomatology classes (*p* = .014), but the odds ratios of the High-Symptomatology and the Low Substance Use with Intermediate Symptom classes were not significantly different from each other (*p* = .496). See Table 5 for the parameter estimates of each nested regression model.

**Table 5.** Odds Ratios and 95% Confidence Intervals for the Logistics Regressions Which Predict Substance use Disorder Diagnoses at the 12-Month Study Visit Based on the Latent Classes Derived from the Baseline Study Visit.

	Model 1			Model 2			Model 3			Model 4		
	*OR*	*95% CI LL*	*95% CI UL*	*OR*	*95% CI LL*	*95% CI UL*	*OR*	*95% CI LL*	*95% CI UL*	*OR*	*95% CI LL*	*95% CI UL*
Latent classes												
Low Symptomatology	Ref	Ref	Ref	Ref	Ref	Ref	Ref	Ref	Ref	Ref	Ref	Ref
Low Substance Use-Intermediate Symptoms	0.99	0.45	2.17	0.86	0.36	2.04	0.66	0.26	1.65	0.62	0.24	1.60
High Substance Use-Intermediate Symptoms	**11.41**	**6.05**	**21.54**	**3.33**	**1.56**	**7.12**	**2.97**	**1.36**	**6.50**	**2.48**	**1.10**	**5.60**
High Symptomatology	**5.41**	**2.87**	**10.20**	1.26	0.45	3.51	0.94	0.32	2.78	0.84	0.27	2.60
Baseline PTSD diagnosis				1.03	0.52	2.03	0.96	0.48	1.92	0.94	0.46	1.92
Baseline SUD diagnosis				**11.40**	**6.72**	**19.35**	**10.77**	**6.28**	**18.48**	**11.29**	**6.26**	**20.36**
Criterion A lifetime trauma				**1.13**	**1.03**	**1.24**	**1.11**	**1.01**	**1.22**	1.08	0.98	1.20
Criterion A trauma exposure between study visits							**1.18**	**1.06**	**1.31**	**1.18**	**1.05**	**1.32**
Psychotropic medication between study visits							1.14	0.65	2.00	1.22	0.68	2.19
Psychotherapy between study visits							1.62	0.78	3.36	1.69	0.77	3.71
Psychiatric hospitalization between study visits							0.88	0.48	1.59	0.96	0.52	1.78
Age										**1.25**	**1.08**	**1.44**
Sex												
Female										Ref	Ref	Ref
Male										1.05	0.57	1.91
Race												
White										Ref	Ref	Ref
Black										1.19	0.49	2.91
Other minority										1.41	0.56	3.60
Multiracial										1.72	0.78	3.79
Hispanic										0.89	0.50	1.56
Household income												
< $25,000										Ref	Ref	Ref
$25,000 - $99,999										1.91	0.92	3.98
> $99,999										1.50	0.63	3.59
Family history												
Psychiatric disorders										0.94	0.51	1.73
Substance use disorders										1.23	0.63	2.42

Model comparison	*F*	*df1*	*df2*	*p*								
Model 1 v 0	**28.56**	**3**	**1025**	**< .001**								
Model 2 v 1	**27.88**	**3**	**1022**	**< .001**								
Model 3 v 2	**2.99**	**4**	**1018**	.**018**								
Model 4 v 3	1.38	10	1008	.186								

Two sets of sensitivity analyses were pursued to assess the robustness of the results. First, we pursued sensitivity analyses in which the predictive modeling was executed using a 5-class solution instead of the 4-class solution. Largely similar latent classes were observed in both the four- and five-class model solutions (Figures S1-S3). After controlling for covariates (Tables S2-S3), the latent classes characterized by interpersonal violence exposure or polysubstance use, respectively, were at elevated risk for PTSD and SUD diagnoses at the 12-month follow-up visit. Second, we executed a latent profile analysis (LPA) to determine if the results were a product of the analytic choice to dichotomize indicator variables (details presented in the Supplemental Methods and Results). In brief, the same indicator variables that were used for the LCA were used for the LPA, except the data were maintained as continuous. Relative model fit statistics evidenced five profiles as an optimal model solution (Tables S3 and S4). The composition of the various latent profiles closely mirrored the previously described latent classes (see Figures S4-S6). In the predictive analyses, latent profiles characterized by interpersonal violence exposure carried prospective risk for PTSD. A single latent profile characterized by polysubstance use was associated with risk for SUD at the 12-month follow-up study visit (Tables S5 and S6).

## Discussion

The current study identified patterns of co-occurrence between trauma exposure, PTSD symptoms, and substance use among trauma exposed youth. These latent classes were differentiated based on the type and pervasiveness of trauma exposure, prevalence of PTSD symptoms, and patterns of substance use. Specifically, trauma was differentiated based on non-interpersonal versus interpersonal traumas, PTSD symptoms were differentiated based on the number of symptoms endorsed, and substance use differentiated based on the propensity for polysubstance use. The patterns of co-occurrence between trauma exposure, PTSD symptoms, and substance use at the baseline study visit corresponded to differences in 12-month longitudinal risk for PTSD and SUD diagnoses.

The most high-level hypotheses of the current study were supported, such that two classes characterized the extremes ends of the conceptual scales. The Low-Symptomatology class was least likely to endorse PTSD symptoms or engage in substance use and was principally exposed to incidental forms of trauma (such as accidents or serious illness). In contrast, the High-Symptomatology class was likely to endorse a multitude of PTSD symptoms, had a high probability of substance use, and was broadly exposed to interpersonal trauma. These findings corroborate research that shows individuals exposed to interpersonal trauma, especially in the context of poly-victimization, are at increased risk for deleterious developmental outcomes, which are characterized, in part, by an elevated likelihood of PTSD and substance use.^1,7,31^ Likewise, these findings highlight that not everyone who experiences trauma, broadly defined, will experience symptoms of PTSD or engage in substance use.^1,10,20,32^ Indeed, these results emphasize that different types of trauma exposures carry disproportionate risk for deleterious health outcomes.

In addition to classes which characterized the extreme end of the trauma, PTSD, and substance use inventories, we hypothesized that additional latent classes would be differentiated based on the endorsement of specific subsets of items. We identified two classes with similar probabilities of endorsing poly-victimization and PTSD symptoms, but which differed markedly in their patterns of substance use. Whereas the Low Substance Use with Intermediate Symptom class was unlikely to engage in any type of substance use, the High Substance Use with Intermediate Symptom class had the highest propensity to engage in polysubstance use behavior relative to any of the latent classes. These findings demonstrate that similar patterns of trauma exposure can lead to divergent substance use behaviors and that PTSD symptomatology need not be comorbid with substance use.^18,33,34^

Beyond using the LCA to differentiate participants based on commonalities in trauma exposure, PTSD symptoms, and substance use, we also sought to determine if the latent classes had predictive validity for PTSD and SUD diagnoses. The three latent classes which tended to endorse PTSD symptoms at the baseline study visit were more likely to have a PTSD diagnosis at the 12-month study visit. Importantly, the classes with prospective risk for PTSD all had higher probabilities of endorsing interpersonal traumas compared to Low-Symptomatology class, findings which map onto literature which highlights that interpersonal traumas tend to be more tightly associated with PTSD symptomatology and psychiatric diagnoses compared to incidental traumas.^1,7,31^ In fact, these findings indicate that adolescents whose trauma history is principally composed of incidental traumas are at low prospective risk for PTSD.

Only the High Substance Use with Intermediate Symptom class was at an elevated risk of SUD at the 12-month study visit. This is notable because compared to the other class which exhibited high rates of polysubstance use, the High-Symptomology class, the High Substance Use with Intermediate Symptom class had lower probabilities of endorsing any given trauma exposure or PTSD symptom. It is possible that the High-Symptomatology class may have captured youth who were experiencing more distress, possibly acutely, and that youth who show some PTSD symptom remission may evolve into either of the Intermediate Symptom classes, contingent on multifactorial predispositions towards substance use.^32,35^ Alternatively, the disparity in longitudinal risk for substance use may be related to more extensive polysubstance use in High Substance Use with Intermediate Symptom class, an interpretation consistent with existing adolescent data.^36^ More generally, the current findings highlight polysubstance use as a developmentally appropriate risk factor for identifying SUD during adolescence.^10,36^

These results suggest that constellations of trauma, PTSD symptoms, and substance use can provide insight into an adolescent's risk for 12-month PTSD and SUD diagnoses above and beyond the risk conferred by demographic characteristics, pre-existing clinical diagnoses, and treatment interventions. Specifically, adolescents who are primarily exposed to incidental trauma and are not engaged in polysubstance are at low risk for future PTSD or SUD diagnoses, whereas adolescents with interpersonal violence exposure or a history of polysubstance use are at elevated risk of PTSD or SUD diagnoses. The recognition of specific ‘types’ may prove to be a useful heuristic for clinicians when gauging an adolescent's prospective clinical risk, especially when other information is not available.

Several considerations should be highlighted for the interpretation of this work (but also see^37,38^ for a full presentation of considerations that pertain to mixture models). First, the measurement windows differed between the PTSD (ie, previous month) and substance use inventories (ie, previous 12 months). The discordance between measurement periods may obscure our ability to identify latent classes which accurately reflect concurrent symptom presentations. Second, the current study could be extended through latent transition analyses [eg,^35^] which would permit additional tests of latent class validity (ie, through indices of measurement invariance) and facilitate tests of the hypothesis that the latent classes identified herein constitute different phenotypic progressions to or from latent classes with more extreme phenotypes. Finally, although this study identified latent classes consistent with previous mixture models performed on single conceptual inventories [eg,^7,10,13^], work with other large cohorts is necessary to determine how generalizable latent class solutions are when trauma, PTSD symptom, and substance use inventories are jointly modeled.

## Supplemental Material

sj-docx-1-css-10.1177_24705470251350144 - Supplemental material for Latent Classes of Adolescent Trauma Exposure, Posttraumatic Stress Disorder Symptoms, and Substance Use Predict Clinical Diagnoses at 12-Month Follow-Up Supplemental material, sj-docx-1-css-10.1177_24705470251350144 for Latent Classes of Adolescent Trauma Exposure, Posttraumatic Stress Disorder Symptoms, and Substance Use Predict Clinical Diagnoses at 12-Month Follow-Up by John Leri, Josh M. Cisler, Shaunna L. Clark, Cody G. Dodd, Saman Siddiqui, Leslie Taylor, Alexa Ayala, Sunita Stewart, Robyn Richmond, Jeffrey D. Shahidullah, Justin F. Rousseau, John M. Hettema, D. Jeffrey Newport, Karen D. Wagner and Charles B. Nemeroff in Chronic Stress
