# Supplementary material for: Latent Classes of Adolescent Trauma Exposure, Posttraumatic Stress Disorder Symptoms, and Substance Use Predict Clinical Diagnoses at 12-Month Follow-Up
Source: Chronic Stress (Thousand Oaks). 2025 Jun 20;9:24705470251350144. doi: 10.1177/24705470251350144 (PMC12182625; doi:10.1177/24705470251350144)
Supplement: sj-docx-1-css-10.1177_24705470251350144 - Supplemental material for Latent Classes of Adolescent Trauma Exposure, Posttraumatic Stress Disorder Symptoms, and Substance Use Predict Clinical Diagnoses at 12-Month Follow-Up [file sj-docx-1-css-10.1177_24705470251350144.docx]

**Table S1.** Items which comprise the trauma inventory

| 1. Have you ever been in a really bad accident, like a car accident, a fall, or a fire? |
| --- |
| 1. Have you ever seen a really bad accident that you weren’t actually in? |
| 1. Have you ever been in a *really* bad storm, like a tornado, a hurricane, or a blizzard? Or in a flood or an earthquake? Or were you ever hit by lightning? |
| 1. Have you ever known someone who got severely hurt or sick, or even died? |
| 1. Have you ever had to stay overnight at the hospital or have an operation? |
| 1. Have you ever had to go away from your parents or family for a long time? Like going to live with another family, or a boarding school or camp, or a hospital or detention center? Or did your mother, father, or someone else who looks after you ever go away for a long time? |
| 1. Have you ever been attacked by a dog or another animal? |
| 1. Has someone ever attacked you or tried to severely hurt you on purpose—like beating, shaking, biting, burning or choking you, or stabbing you with a knife or shooting you with a gun? Or has anyone ever punished you so hard that you were hurt really badly or had to go to the doctor or hospital-like a spanking, whipping, or beating? |
| 1. Has someone ever *told* you they were going to hurt you really badly, or *acted like* they would hurt you really badly? |
| 1. Has someone a lot older ever tried to steal from you? Or from a family member or friend when you were right there? // Has someone ever mugged you or held you up to try to steal from you? Or have you ever been present when a family member or close friend was mugged? |
| 1. Has someone ever kidnapped you or taken you away when they weren’t supposed to? Or has someone in your family or a close friend ever been kidnapped? |
| 1. Have you ever seen people in your family fighting or attacking each other? Or shooting with a gun? Or stabbing with a knife? Or beating each other up? |
| 1. Even if they weren't physically attacking each other, have you ever heard people in your family really yelling and screaming at each other a lot? |
| 1. Has someone in your family ever been put in jail or prison? Or have the police or soldiers ever come to your house and said you or your family were in big trouble? |
| 1. Have you ever seen people outside your home fighting or attacking each other? Or shooting with a gun? Or stabbing with a knife? Or beating each other up? |
| 1. Even if they weren't physically attacking each other, have you ever heard people outside your home really yelling and screaming at each other a lot? |
| 1. Have you seen or heard people attacking each other *for real* on television, radio, or online? Like a war or a building blowing up? |
| 1. Has someone ever touched your body in a way you didn’t want them to or in a way that made you uncomfortable? |
| 1. Have you ever been picked on by someone over and over again? This type of bullying includes someone calling you names, making fun of you, spreading lies or rumors about you, telling other people not to like you, leaving you out of activities, or saying mean things to hurt your feelings. |
| 1. Has someone ever used social media, text messaging, or group chats to bully you?  This can include someone spreading rumors about you online, sending you mean messages, pretending to be you online, or posting messages or videos to embarrass you. |

*Note*. All items are from the Traumatic Events Screening Inventory (TESI-C) except for items and #19 and #20 (i.e., bullying items). The two bullying items were added by the Texas Childhood Trauma Research Network study team.

**Figure S1**. Probability of endorsing trauma items by latent class membership for the 5-class solution


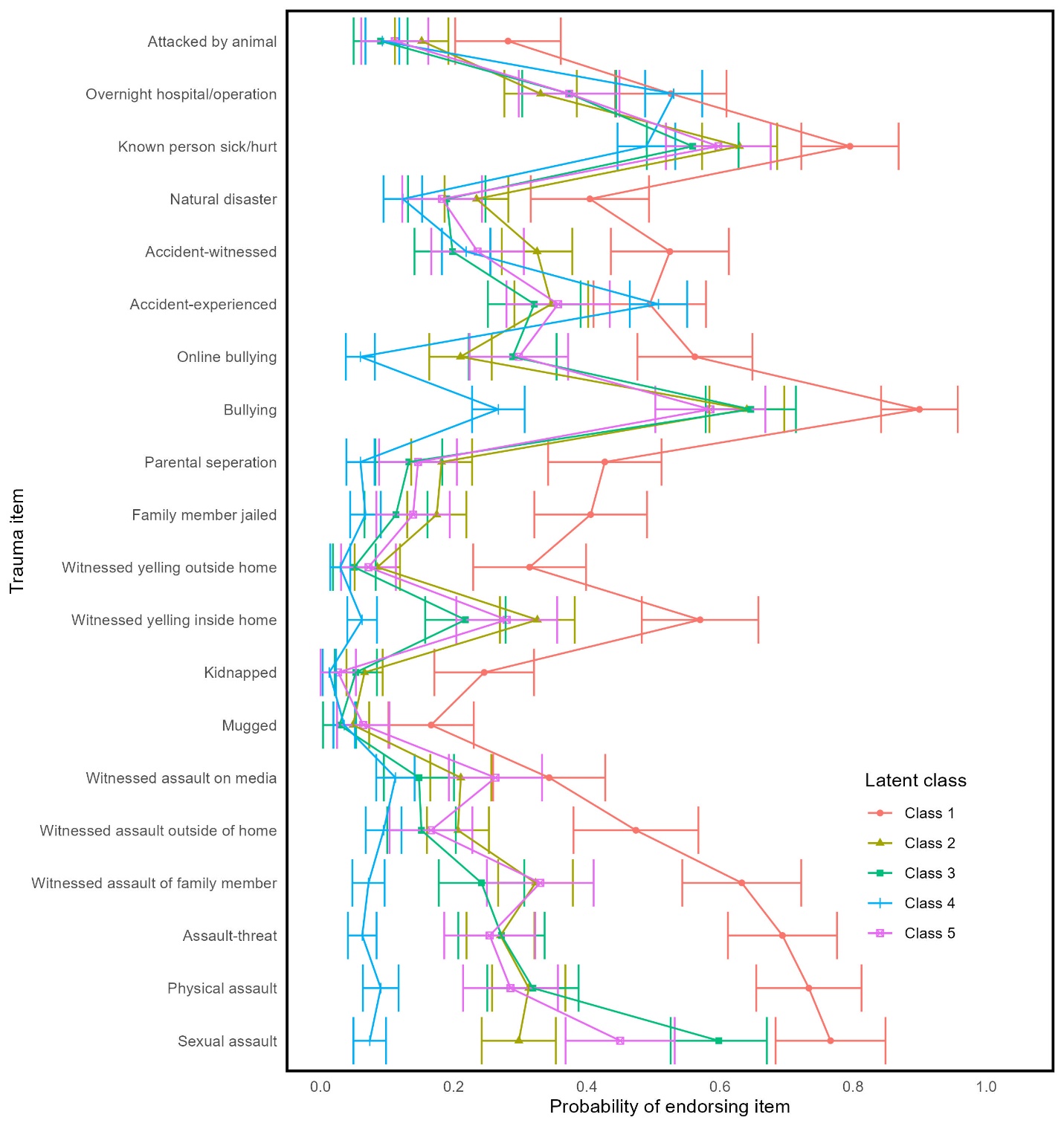


**Figure S2**. Probability of endorsing a posttraumatic stress disorder symptom by latent class membership for the 5-class solution


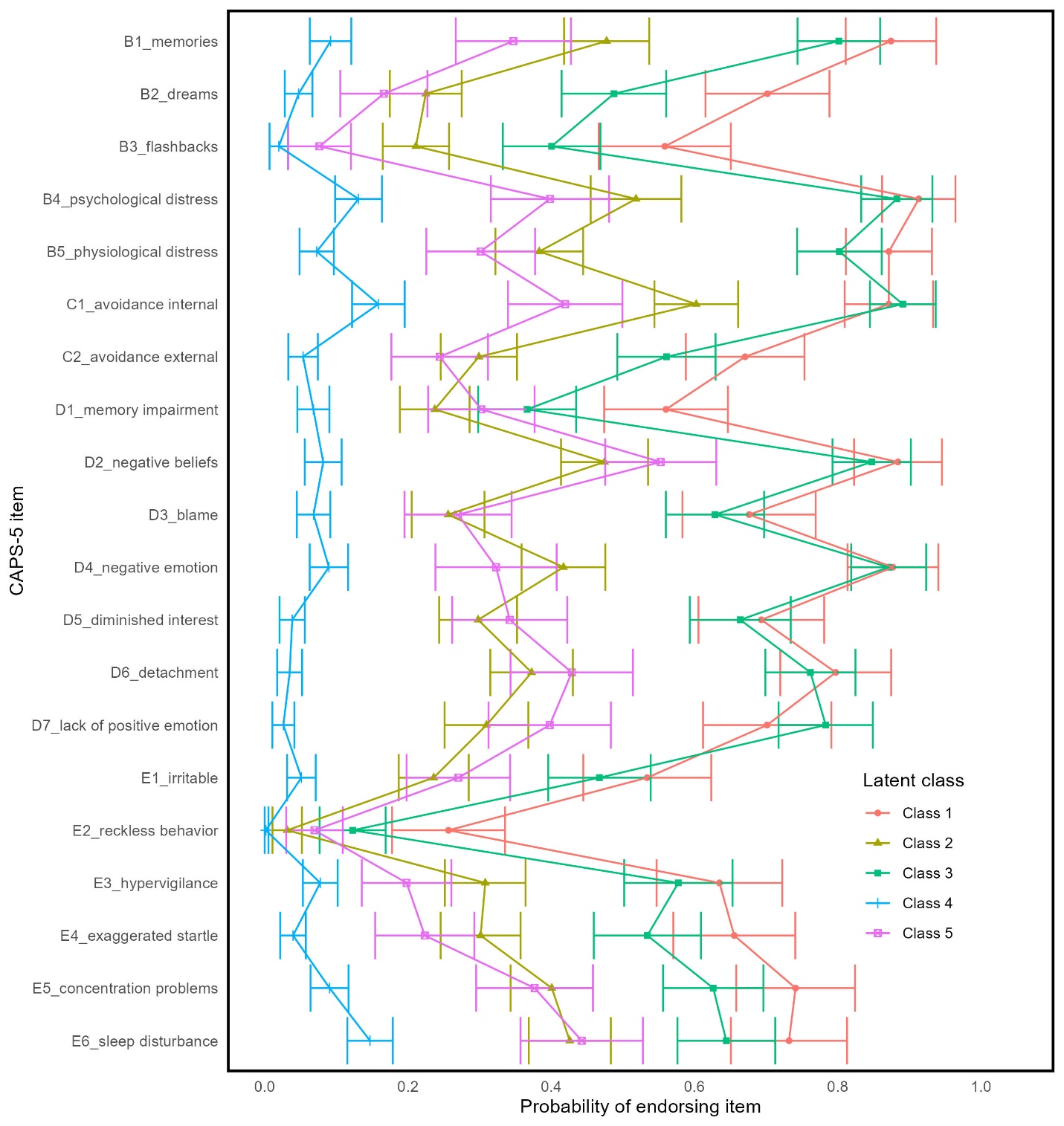


**Figure S3**. Probability of endorsing substance use items by latent class membership for the 5-class solution


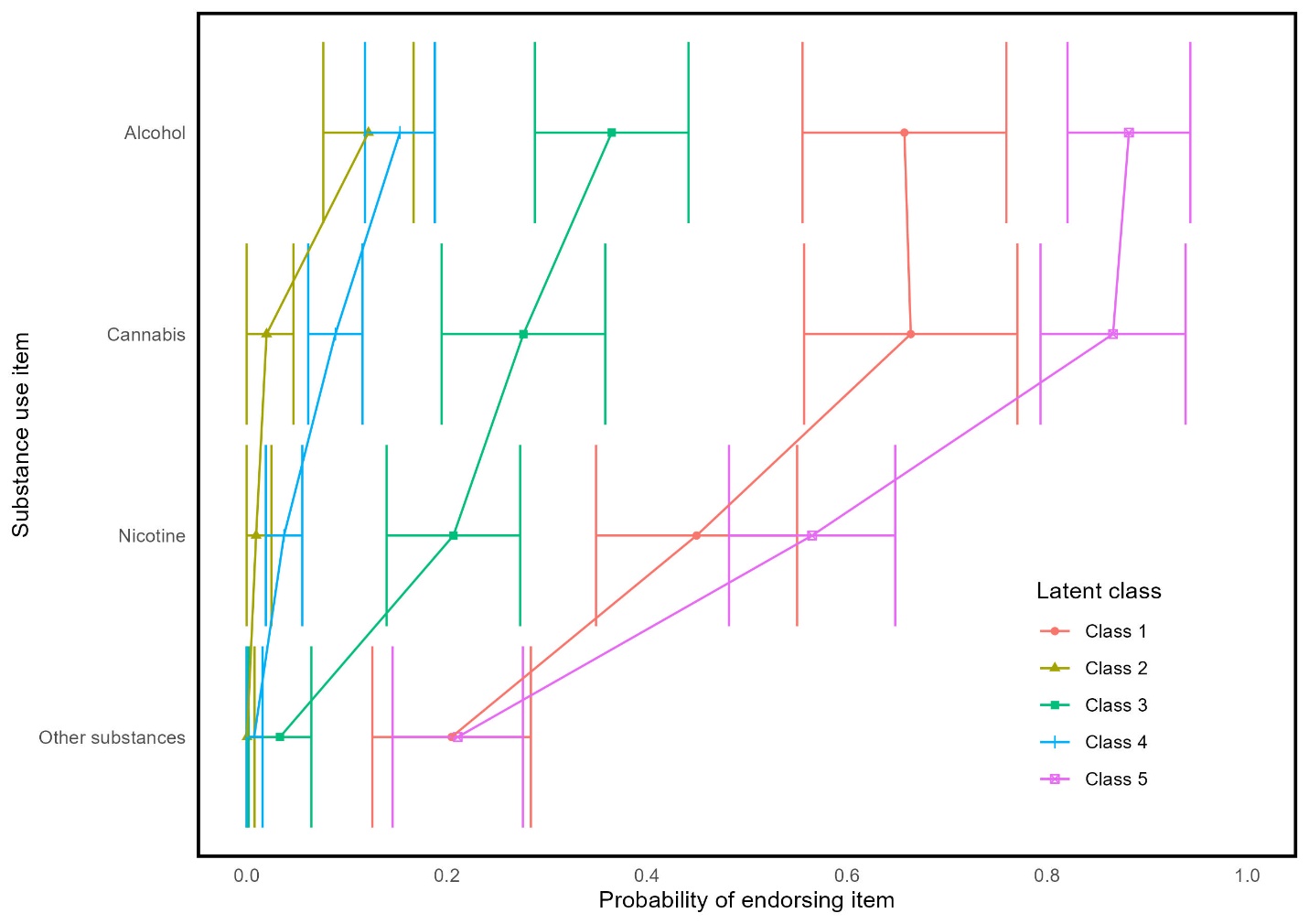


**Table S2**. Unstandardized regression coefficients from the logistics regressions which predict PTSD diagnoses at the 12-month study visits based on latent class groupings from the baseline study visit: 5-class solution

|  | | | Model 1 | | | | | Model 2 | | | | | | Model 3 | | | | | | Model 4 | | | | |
| --- | --- | --- | --- | --- | --- | --- | --- | --- | --- | --- | --- | --- | --- | --- | --- | --- | --- | --- | --- | --- | --- | --- | --- | --- |
|  | | | *OR* | | *95% CI LL* | *95% CI UL* | | *OR* | | *95% CI LL* | | *95% CI UL* | | *OR* | | *95% CI LL* | | *95% CI UL* | | *OR* | | *95% CI LL* | | *95% CI UL* |
| Latent classes | | |  | |  |  | |  | |  | |  | |  | |  | |  | |  | |  | |  |
| LC4 | | | Ref | | Ref | Ref | | Ref | | Ref | | Ref | | Ref | | Ref | | Ref | | Ref | | Ref | | Ref |
| LC1 | | | **27.13** | | **9.88** | **74.48** | | **9.50** | | **2.58** | | **34.94** | | **5.53** | | **1.45** | | **21.14** | | **5.80** | | **1.45** | | **23.20** |
| LC2 | | | **7.76** | | **2.83** | **21.27** | | **5.94** | | **2.10** | | **16.82** | | **3.83** | | **1.34** | | **10.94** | | **3.97** | | **1.36** | | **11.57** |
| LC3 | | | **17.23** | | **6.45** | **46.05** | | **6.86** | | **2.28** | | **20.60** | | **4.26** | | **1.40** | | **12.96** | | **4.43** | | **1.42** | | **13.89** |
| LC5 | | | **10.99** | | **3.93** | **30.71** | | **7.53** | | **2.53** | | **22.43** | | **5.25** | | **1.74** | | **15.90** | | **5.81** | | **1.83** | | **18.43** |
| Baseline PTSD diagnosis | | |  | |  |  | | **3.11** | | **1.82** | | **5.32** | | **3.13** | | **1.78** | | **5.50** | | **3.26** | | **1.82** | | **5.84** |
| Baseline SUD diagnosis | | |  | |  |  | | 1.42 | | 0.84 | | 2.38 | | 1.15 | | 0.66 | | 2.01 | | 1.04 | | 0.58 | | 1.87 |
| Criterion A lifetime trauma | | |  | |  |  | | 0.99 | | 0.91 | | 1.08 | | 0.95 | | 0.86 | | 1.04 | | 0.95 | | 0.86 | | 1.05 |
| Criterion A trauma exposure between study visits | | |  | |  |  | |  | |  | |  | | **1.26** | | **1.15** | | **1.39** | | **1.30** | | **1.18** | | **1.44** |
| Psychotropic medication between study visits | | |  | |  |  | |  | |  | |  | | **1.71** | | **1.08** | | **2.73** | | **1.86** | | **1.14** | | **3.04** |
| Psychotherapy between study visits | | |  | |  |  | |  | |  | |  | | 1.81 | | 0.90 | | 3.66 | | 1.81 | | 0.87 | | 3.77 |
| Psychiatric hospitalization between study visits | | |  | |  |  | |  | |  | |  | | 1.19 | | 0.72 | | 1.98 | | 1.14 | | 0.68 | | 1.93 |
| Age | | |  | |  |  | |  | |  | |  | |  | |  | |  | | 0.99 | | 0.88 | | 1.11 |
| Male | | |  | |  |  | |  | |  | |  | |  | |  | |  | | 1.14 | | 0.68 | | 1.89 |
| Race | | |  | |  |  | |  | |  | |  | |  | |  | |  | |  | |  | |  |
| White | | |  | |  |  | |  | |  | |  | |  | |  | |  | | Ref | | Ref | | Ref |
| Black | | |  | |  |  | |  | |  | |  | |  | |  | |  | | 0.59 | | 0.26 | | 1.34 |
| Other minority | | |  | |  |  | |  | |  | |  | |  | |  | |  | | 0.84 | | 0.36 | | 1.96 |
| Multiracial | | |  | |  |  | |  | |  | |  | |  | |  | |  | | 1.55 | | 0.82 | | 2.92 |
| Hispanic | | |  | |  |  | |  | |  | |  | |  | |  | |  | | 1.42 | | 0.86 | | 2.33 |
| Household income | | |  | |  |  | |  | |  | |  | |  | |  | |  | |  | |  | |  |
| < $25,000 | | |  | |  |  | |  | |  | |  | |  | |  | |  | | Ref | | Ref | | Ref |
| $25,000 - $99,999 | | |  | |  |  | |  | |  | |  | |  | |  | |  | | 0.79 | | 0.44 | | 1.45 |
| > $99,999 | | |  | |  |  | |  | |  | |  | |  | |  | |  | | 1.31 | | 0.65 | | 2.65 |
| Family history | | |  | |  |  | |  | |  | |  | |  | |  | |  | |  | |  | |  |
| Psychiatric disorders | | |  | |  |  | |  | |  | |  | |  | |  | |  | | 0.98 | | 0.59 | | 1.65 |
| Substance use disorders | | |  | |  |  | |  | |  | |  | |  | |  | |  | | 0.93 | | 0.55 | | 1.57 |
| Model comparison | *F* | *df1* | | *df2* | | | *p* | |  | |  | |  | |  | |  | |  | |  | |  | |
| Model 1 v 0 | **14.00** | **4** | | **1024** | | | **< .001** | |  | |  | |  | |  | |  | |  | |  | |  | |
| Model 2 v 1 | **6.55** | **3** | | **1021** | | | **< .001** | |  | |  | |  | |  | |  | |  | |  | |  | |
| Model 3 v 2 | **9.04** | **4** | | **1017** | | | **< .001** | |  | |  | |  | |  | |  | |  | |  | |  | |
| Model 4 v 3 | 1.11 | 10 | | 1007 | | | .348 | |  | |  | |  | |  | |  | |  | |  | |  | |

**Table S3**. Unstandardized regression coefficients from the logistics regressions which predict SUD diagnoses at the 12-month study visits based on latent class groupings from the baseline study visit: 5-class solution

|  | | | Model 1 | | | | | Model 2 | | | | | | Model 3 | | | | | | Model 4 | | | | |
| --- | --- | --- | --- | --- | --- | --- | --- | --- | --- | --- | --- | --- | --- | --- | --- | --- | --- | --- | --- | --- | --- | --- | --- | --- |
|  | | | *OR* | | *95% CI LL* | *95% CI UL* | | *OR* | | *95% CI LL* | | *95% CI UL* | | *OR* | | *95% CI LL* | | *95% CI UL* | | *OR* | | *95% CI LL* | | *95% CI UL* |
| Latent classes | | |  | |  |  | |  | |  | |  | |  | |  | |  | |  | |  | |  |
| LC4 | | | Ref | | Ref | Ref | | Ref | | Ref | | Ref | | Ref | | Ref | | Ref | | Ref | | Ref | | Ref |
| LC1 | | | **10.57** | | **5.21** | **21.44** | | 1.85 | | 0.54 | | 6.35 | | 1.56 | | 0.43 | | 5.65 | | 1.26 | | 0.32 | | 4.99 |
| LC2 | | | 1.35 | | 0.60 | 3.06 | | 1.19 | | 0.48 | | 2.94 | | 0.96 | | 0.37 | | 2.48 | | 0.89 | | 0.33 | | 2.40 |
| LC3 | | | 1.94 | | 0.87 | 4.31 | | 1.00 | | 0.36 | | 2.79 | | 0.82 | | 0.28 | | 2.37 | | 0.78 | | 0.26 | | 2.32 |
| LC5 | | | **13.53** | | **6.92** | **26.45** | | **4.03** | | **1.82** | | **8.94** | | **3.64** | | **1.61** | | **8.23** | | **2.93** | | **1.24** | | **6.91** |
| Baseline PTSD diagnosis | | |  | |  |  | | 1.09 | | 0.55 | | 2.17 | | 0.97 | | 0.48 | | 1.96 | | 0.94 | | 0.45 | | 1.94 |
| Baseline SUD diagnosis | | |  | |  |  | | **11.43** | | **6.66** | | **19.63** | | **10.85** | | **6.26** | | **18.82** | | **11.49** | | **6.32** | | **20.90** |
| Criterion A lifetime trauma | | |  | |  |  | | 1.10 | | 0.99 | | 1.23 | | 1.07 | | 0.96 | | 1.20 | | 1.06 | | 0.94 | | 1.19 |
| Criterion A trauma exposure between study visits | | |  | |  |  | |  | |  | |  | | **1.17** | | **1.04** | | **1.31** | | **1.17** | | **1.04** | | **1.31** |
| Psychotropic medication between study visits | | |  | |  |  | |  | |  | |  | | 1.10 | | 0.63 | | 1.94 | | 1.19 | | 0.65 | | 2.16 |
| Psychotherapy between study visits | | |  | |  |  | |  | |  | |  | | 1.62 | | 0.78 | | 3.35 | | 1.67 | | 0.76 | | 3.69 |
| Psychiatric hospitalization between study visits | | |  | |  |  | |  | |  | |  | | 0.84 | | 0.45 | | 1.54 | | 0.91 | | 0.48 | | 1.72 |
| Age | | |  | |  |  | |  | |  | |  | |  | |  | |  | | **1.24** | | **1.07** | | **1.43** |
| Male | | |  | |  |  | |  | |  | |  | |  | |  | |  | | 1.07 | | 0.58 | | 1.95 |
| Race | | |  | |  |  | |  | |  | |  | |  | |  | |  | |  | |  | |  |
| White | | |  | |  |  | |  | |  | |  | |  | |  | |  | | Ref | | Ref | | Ref |
| Black | | |  | |  |  | |  | |  | |  | |  | |  | |  | | 1.20 | | 0.49 | | 2.94 |
| Other minority | | |  | |  |  | |  | |  | |  | |  | |  | |  | | 1.40 | | 0.55 | | 3.54 |
| Multiracial | | |  | |  |  | |  | |  | |  | |  | |  | |  | | 1.66 | | 0.75 | | 3.66 |
| Hispanic | | |  | |  |  | |  | |  | |  | |  | |  | |  | | 0.90 | | 0.51 | | 1.58 |
| Household income | | |  | |  |  | |  | |  | |  | |  | |  | |  | |  | |  | |  |
| < $25,000 | | |  | |  |  | |  | |  | |  | |  | |  | |  | | Ref | | Ref | | Ref |
| $25,000 - $99,999 | | |  | |  |  | |  | |  | |  | |  | |  | |  | | 1.95 | | 0.94 | | 4.07 |
| > $99,999 | | |  | |  |  | |  | |  | |  | |  | |  | |  | | 1.53 | | 0.64 | | 3.65 |
| Family history | | |  | |  |  | |  | |  | |  | |  | |  | |  | |  | |  | |  |
| Psychiatric disorders | | |  | |  |  | |  | |  | |  | |  | |  | |  | | 0.98 | | 0.53 | | 1.81 |
| Substance use disorders | | |  | |  |  | |  | |  | |  | |  | |  | |  | | 1.18 | | 0.60 | | 2.32 |
| Model comparison | *F* | *df1* | | *df2* | | | *p* | |  | |  | |  | |  | |  | |  | |  | |  | |
| Model 1 v 0 | **25.25** | **4** | | **1024** | | | **< .001** | |  | |  | |  | |  | |  | |  | |  | |  | |
| Model 2 v 1 | **25.91** | **3** | | **1021** | | | **< .001** | |  | |  | |  | |  | |  | |  | |  | |  | |
| Model 3 v 2 | 2.67 | 4 | | 1017 | | | .475 | |  | |  | |  | |  | |  | |  | |  | |  | |
| Model 4 v 3 | 1.27 | 10 | | 1007 | | | .501 | |  | |  | |  | |  | |  | |  | |  | |  | |

**Details about Latent Profile Sensitivity Analyses**

This section provides an abbreviated method (does not repeat information which is redundant with primary manuscript text) and results section for latent profile analysis (LPA). LPA was conducted to determine if the latent classes described in the manuscript were a spurious result related to the use of binary indicator variables.

**Supplemental Methods**

**Measures**

**Trauma exposure**

Trained interviewers administered the Traumatic Events Screening Inventory for Children (TESI-C) to measure youth’s exposure to 20 types of trauma experiences. Youth’s descriptions of each exposure were used to determine if the exposure qualified as a DSM-5 PTSD criterion A exposure. Item responses were maintained if the exposure met criterion A and were coded as no exposure if the exposure did not meet criterion A. Frequency of exposure was indexed for each exposure that youth endorsed. Contingent on the item, frequency of exposure was indexed with one of two schemes: (A) 1 = only once, 2 = few times, 3 = many times; (B) 1 = only once, 2 = few times, 3 = several times per month, 4 = several times per week.

**Posttraumatic stress disorder**

Trained interviewers administered the Clinician-Administered PTSD Scale for DSM-5 Child/Adolescent Version (CAPS-CA-5) to youth [24]. Interviewers conducted a structured interview that produced severity scores for the 20 items constituting the re-experiencing, avoidance, negative alterations in cognition and mood, and arousal and reactivity symptom clusters of DSM-5 PTSD. Item severity scores could range from 0 to 4, 0 = absent, 1 = mild/subthreshold, 2 = moderate/threshold, 3 = severe/markedly elevated, 4 = extreme/incapacitating. PTSD diagnostic status was determined according to the publisher’s directions.

**Substance use**

Four items from the CRAFFT 2.1+N (Car, Relax, Alone, Forget, Friends, Trouble: Version 2.1 + Nicotine; [25]) were used to assess substance use. Youth self-reported the number of days on which they consumed alcohol, cannabis products, nicotine, or other drugs during the previous 12-months. The number of consumption days was recoded such that 0 = 0 consumption days, 1 = 1 to 10 consumption days, 2 = 11 to 30 consumption days, 3 = more than 30 consumption days. Diagnoses for alcohol use disorder and substance use disorder were derived from the Mini-International Neuropsychiatric Interview for Children and Adolescents (MINI-KID; [26]), a structured diagnostic interview. A binary substance use disorder indicator variable was created from the MINI-KID diagnoses for which 0 indicated the absence of any type of substance use disorder and 1 indicated the presence of either an alcohol use disorder or non-alcohol substance use disorder.

**Data Analysis**

Statistical analyses were executed using R statistical software, version 4.3.1. The full sample consisted of 2,496 youth (enrollment October 2020 through March 2024). Because there were a limited number of youth who endorsed substance use before age 13, the analytical sample was restricted to youth ages 13 through 20 (*n* = 1,826). Latent profile analyses (LPA) were executed using the ‘mclust’ package as implemented in the ‘tidyLPA’ package. A total of 44 continuous indicators from the baseline study visit (20 trauma indicators, 20 PTSD indicators, and 4 substance use indicators) were used as input for the LPA. A total of 1,291 participants had complete data for LPA model enumeration. Data was imputed with a random-forest algorithm for participants who had missing data for the baseline indicator variables; an imputation which was only used for LPA model enumeration. LPA solutions were enumerated via an expectation maximization algorithm for 1 through 10 classes. All combinations of parameterizations for the within-group covariance matrices were executed. The most parsimonious parameterizations were profiles which had spherical distributions, equal volumes, and equal shapes; which were used for the reported model solutions. Model fit statistics, including Akaike information criterion (AIC), Bayesian information criterion (BIC), and the bootstrapped likelihood ratio test (BLRT), were used to compare the relative fit of the LPA solutions. Entropy was used to assess the differentiation between LPA classes for each model solution. Additional considerations for selecting an optimal number of classes included the proportion of youth assigned to each class and the conceptual interpretability of each class.

After an optimal number of latent profiles were identified, participants were assigned to the latent profiles with the highest posterior probability. Latent profile assignments were then used to predict SUD and PTSD diagnoses at the 12-month follow-up study visit.

Prior to the predictive analyses, the ‘mice’ package [28] was used to perform multiple imputation for missing covariates from the baseline study visit and selected data elements at the follow-up study visits. Specifically, follow-up data were imputed if participants were lost to follow-up or if the follow-up study visit was completed outside of the accepted range (+- 30 days of the scheduled study visit). Missing data were not imputed for participants who had not matriculated to the follow-up study visits. Logistic regression and predictive mean matching, respectively, were used to impute categorical and continuous variables. After missing data were imputed there were 1,029 participants with clinical diagnostic data at the 12-month study visit.

Nested logistic regressions were used to determine if latent profile membership predicted 12-month PTSD and SUD diagnoses. Each nested regression added a block of variables to the models which predicted 12-month diagnostic status. The null model included only an intercept. Step-1 added the latent profiles to the model. Step-2 added lifetime trauma exposure, PTSD diagnoses, and SUD diagnoses from the baseline study visit. Step-3 added trauma exposure, psychotherapy involvement, psychotropic medication use, and psychiatric hospitalizations which occurred between the baseline study visit and the 12-month study visit. Step-4 added demographic variables including age, sex, race, ethnicity, household income, and family history of psychiatric and substance use disorders. Models were fitted on all imputed datasets (*n* = 100) and parameter estimates were pooled according to Rubin’s rules [29]. The relative fit of the nested models was compared using the multivariate Wald test.

**Supplemental Results**

Model solutions with up to 10 classes were fitted. The five-profile solution was deemed optimal. The optimal number of profiles was determined through two primary considerations, relative model fit statistics and conceptual interpretability. Although there was a general lack of distinction across the model solutions based on relative model fit statistics (i.e., loglikelihood, AIC, BIC), the five-profile solution maximized the entropy values and differentiated the latent profiles in a manner that led to a parsimonious interpretation (Table S3). Additional profiles, although interpretable, added model complexity without contributing unique information. The probabilities of each latent profile endorsing individual trauma, PTSD, and substance use indicators are shown in Figures S4 through S6, respectively.

The two latent profiles which characterized the extreme ends of the conceptual inventories could be described as ‘Low-Symptomatology’ (37%, Profile 4) and ‘High-Symptomatology’ (8%, Profile 3) profiles. Whereas the Low-Symptomatology profile had low probabilities of endorsing most traumas, PTSD symptoms, and substance use indicators, the High-Symptomatology profile had the highest probabilities of endorsing any given trauma, PTSD symptom, or substance use indicator. More concretely, the Low-Symptomatology profile was principally exposed to incidental forms of trauma, carried a low burden for PTSD, and was likely to report alcohol use (if any substance use was reported at all). In contrast, the High-Symptomatology profile was likely to be exposed to a broad range of interpersonal traumas, endorse a broad range of PTSD symptoms, and was likely to be engaged in polysubstance use.

Three latent profiles were identified which characterized intermediate levels of trauma exposure (termed ‘Intermediate Exposure’ profiles). Although all three latent profiles were likely to be exposed to forms of interpersonal trauma, they differentiated based on the propensity to endorse PTSD symptoms and substance use. One of the intermediate trauma exposure profiles carried a high probability of endorsing most PTSD symptoms but exhibited markedly low levels of substance use (12%, Profile 1, Intermediate Exposure with High PTSD Symptomatology). The remaining two intermediate trauma profiles similarly endorsed intermediate levels of PTSD symptomatology, but whereas one profile was unlikely to endorse substance use in significant ways (29%, Profile 2, Intermediate Exposure with Moderate PTSD Symptomatology), the other profile was likely to endorse polysubstance use (13%, Profile 5, Intermediate Exposure with Polysubstance Use).

Logistic regressions were fitted that predicted clinical PTSD diagnoses at the 12-month follow-up visit. Step-1 showed that the latent profiles significantly improved model fit compared to the intercept only model (*F_(4, 1024)_* = 16.34, *p* < .001). Specifically, relative to the Low-Symptomatology profile, the other latent profiles were significantly more likely to have a PTSD diagnosis at the 12-month follow-up visit (odds ratios ranged from 5.66-19.42). Step-2 indicated that lifetime trauma exposure, PTSD diagnoses, and SUD diagnoses from the baseline visit improved model fit (*F_(3, 1021)_* = 5.72, *p* < .001); as did the inclusion of recurrent trauma and psychiatric therapy involvement during the inter-visit interval within Step-3 (*F_(4, 1017)_* = 4.54, *p* = .001). The addition of demographic covariates in Step-4 did not significantly improve model fit when predicting 12-month PTSD diagnoses (*F_(10, 1007)_* = 0.92, *p* = .516). Although the inclusion of covariates attenuated the association between the latent profiles and 12-month PTSD diagnoses (odds ratios ranged from 3.17-5.84 in the fully adjusted model), the Intermediate Exposure and High-Symptomatology latent profiles were all significantly more likely to have a PTSD diagnosis at the 12-month study visit relative to the Low-Symptomatology class. Linear contrasts indicated that, in the fully adjusted model, the odds ratios of 12-month PTSD diagnoses were not significantly different between the Intermediate Exposure and High-Symptomatology latent classes (all *p* >= .319). See Table S5 for the parameter estimates of each nested regression.

Logistic regressions were fitted that predicted clinical SUD diagnoses at the 12-month follow-up visit. Step-1 showed that the latent profiles significantly improved model fit compared to the intercept only model (*F_(4, 1024)_* = 26.83, *p* < .001). Relative to the Low-Symptomatology profile, the Intermediate Exposure with Polysubstance Use (odds ratio = 11.27) and High-Symptomatology (odds ratio = 13.01) latent profiles were significantly more likely to have a SUD diagnosis at the 12-month follow-up visit. Step-2 indicated that lifetime trauma exposure, PTSD diagnoses, and SUD diagnoses from the baseline visit improved model fit (*F_(3, 1021)_* = 17.65, *p* < .001). Neither the addition of recurrent trauma and psychiatric therapy involvement during the inter-visit interval within Step-3 (*F_(4, 1017)_* = 1.44, *p* = .219) nor the addition of demographic covariates in Step-4 significantly improve model fit when predicting 12-month SUD diagnoses (*F_(10, 1007)_* = 1.26, *p* = .249). The inclusion of covariates attenuated the association between the Intermediate Exposure with Polysubstance Use profile and 12-month SUD diagnosis (odds ratio = 2.47) and nullified the association between the High-Symptomatology profile and 12-month SUD diagnoses. Linear contrasts indicated that, in the fully adjusted model, that the odds ratio of the Intermediate Exposure with Polysubstance Use profile was larger than all the other profiles (all *p* <= .003) except the High Symptomology class (*p* = .237). See Table S6 for the parameter estimates of each nested regression model.

**Table S3**. Model fit statistics per each n-profile model solution

| N-profiles | Loglikelihood | AIC | BIC | Entropy | BLRT-*p* |
| --- | --- | --- | --- | --- | --- |
| 1 | -105586 | 211348 | 211832 | 1.00 | - |
| 2 | -100197 | 200659 | 201392 | 0.93 | .010 |
| 3 | -98882 | 198121 | 199102 | 0.91 | .010 |
| 4 | -98188 | 196821 | 198050 | 0.91 | .010 |
| 5 | -97455 | 195445 | 196922 | 0.93 | .010 |
| 6 | -97158 | 194942 | 196666 | 0.91 | .010 |
| 7 | -96589 | 193894 | 195867 | 0.92 | .010 |
| 8 | -95898 | 192602 | 194822 | 0.92 | .010 |
| 9 | -96019 | 192934 | 195403 | 0.91 | .634 |
| 10 | -95440 | 191866 | 194583 | 0.93 | .010 |

*Note*. AIC = Akaike information criterion, BIC = Bayesian information criterion, BLRT = bootstrapped likelihood ratio test.

**Table S4**. Proportion of participants in each profile per each n-profile model solution

| N-profiles | Profile 1 | Profile 2 | Profile 3 | Profile 4 | Profile 5 | Profile 6 | Profile 7 | Profile 8 | Profile 9 | Profile 10 |
| --- | --- | --- | --- | --- | --- | --- | --- | --- | --- | --- |
| 1 | 100% | - | - | - | - | - | - | - | - | - |
| 2 | 37% | 63% | - | - | - | - | - | - | - | - |
| 3 | 17% | 40% | 43% | - | - | - | - | - | - | - |
| 4 | 14% | 31% | 41% | 13% | - | - | - | - | - | - |
| 5 | 12% | 29% | 8% | 37% | 13% | - | - | - | - | - |
| 6 | 11% | 17% | 13% | 8% | 37% | 12% | - | - | - | - |
| 7 | 9% | 13% | 18% | 8% | 37% | 2% | 13% | - | - | - |
| 8 | 7% | 7% | 8% | 11% | 5% | 36% | 14% | 12% | - | - |
| 9 | 7% | 13% | 10% | 15% | 5% | 32% | 3% | 12% | 2% | - |
| 10 | 6% | 11% | 9% | 2% | 4% | 34% | 13% | 3% | 12% | 5% |

*Note*. Profile designations are arbitrary across model solutions, which means that the phenotype which describes Profile 1 for the 2-profile solution is not necessarily the same phenotype which is described by Profile 1 in the 3-profile solution.

**Table S5**. Unstandardized regression coefficients from the logistics regressions which predict PTSD diagnoses at the 12-month study visits based on latent profiles from the baseline study visit: 5-class solution

|  | | | Model 1 | | | | | Model 2 | | | | | | Model 3 | | | | | | Model 4 | | | | |
| --- | --- | --- | --- | --- | --- | --- | --- | --- | --- | --- | --- | --- | --- | --- | --- | --- | --- | --- | --- | --- | --- | --- | --- | --- |
|  | | | *OR* | | *95% CI LL* | *95% CI UL* | | *OR* | | *95% CI LL* | | *95% CI UL* | | *OR* | | *95% CI LL* | | *95% CI UL* | | *OR* | | *95% CI LL* | | *95% CI UL* |
| Latent profiles | | |  | |  |  | |  | |  | |  | |  | |  | |  | |  | |  | |  |
| Low-Symptomatology | | | Ref | | Ref | Ref | | Ref | | Ref | | Ref | | Ref | | Ref | | Ref | | Ref | | Ref | | Ref |
| Intermediate Exposure-High PTSD | | | **17.79** | | **7.45** | **42.47** | | **6.87** | | **2.40** | | **19.65** | | **5.58** | | **1.96** | | **15.83** | | **5.84** | | **1.95** | | **17.43** |
| Intermediate Exposure-Moderate PTSD | | | **5.66** | | **2.48** | **12.95** | | **3.79** | | **1.56** | | **9.20** | | **3.06** | | **1.27** | | **7.36** | | **3.17** | | **1.28** | | **7.90** |
| Intermediate Exposure-High SU | | | **7.04** | | **2.87** | **17.29** | | **4.65** | | **1.70** | | **12.72** | | **4.12** | | **1.50** | | **11.34** | | **4.65** | | **1.60** | | **13.56** |
| High-Symptomatology | | | **19.42** | | **7.73** | **48.77** | | **5.78** | | **1.77** | | **18.87** | | **4.27** | | **1.27** | | **14.39** | | **4.19** | | **1.18** | | **14.91** |
| Baseline PTSD diagnosis | | |  | |  |  | | **3.00** | | **1.76** | | **5.11** | | **3.04** | | **1.75** | | **5.28** | | **3.21** | | **1.82** | | **5.68** |
| Baseline SUD diagnosis | | |  | |  |  | | 1.48 | | 0.80 | | 2.74 | | 1.35 | | 0.71 | | 2.59 | | 1.27 | | 0.65 | | 2.47 |
| Criterion A lifetime trauma | | |  | |  |  | | 1.00 | | 0.93 | | 1.07 | | 0.97 | | 0.89 | | 1.05 | | 0.97 | | 0.89 | | 1.05 |
| Criterion A trauma exposure between study visits | | |  | |  |  | |  | |  | |  | | **1.23** | | **1.12** | | **1.36** | | **1.26** | | **1.13** | | **1.40** |
| Psychotropic medication between study visits | | |  | |  |  | |  | |  | |  | | **1.68** | | **1.01** | | **2.80** | | **1.75** | | **1.02** | | **2.99** |
| Psychotherapy between study visits | | |  | |  |  | |  | |  | |  | | 0.98 | | 0.50 | | 1.93 | | 0.93 | | 0.46 | | 1.87 |
| Psychiatric hospitalization between study visits | | |  | |  |  | |  | |  | |  | | 1.01 | | 0.60 | | 1.69 | | 0.98 | | 0.58 | | 1.67 |
| Age | | |  | |  |  | |  | |  | |  | |  | |  | |  | | 0.97 | | 0.86 | | 1.09 |
| Male | | |  | |  |  | |  | |  | |  | |  | |  | |  | | 1.09 | | 0.63 | | 1.91 |
| Race | | |  | |  |  | |  | |  | |  | |  | |  | |  | |  | |  | |  |
| White | | |  | |  |  | |  | |  | |  | |  | |  | |  | | Ref | | Ref | | Ref |
| Black | | |  | |  |  | |  | |  | |  | |  | |  | |  | | 0.59 | | 0.24 | | 1.41 |
| Other minority | | |  | |  |  | |  | |  | |  | |  | |  | |  | | 0.58 | | 0.24 | | 1.43 |
| Multiracial | | |  | |  |  | |  | |  | |  | |  | |  | |  | | 1.49 | | 0.76 | | 2.91 |
| Hispanic | | |  | |  |  | |  | |  | |  | |  | |  | |  | | 1.25 | | 0.75 | | 2.08 |
| Household income | | |  | |  |  | |  | |  | |  | |  | |  | |  | |  | |  | |  |
| < $25,000 | | |  | |  |  | |  | |  | |  | |  | |  | |  | | Ref | | Ref | | Ref |
| $25,000 - $99,999 | | |  | |  |  | |  | |  | |  | |  | |  | |  | | 0.83 | | 0.45 | | 1.53 |
| > $99,999 | | |  | |  |  | |  | |  | |  | |  | |  | |  | | 1.22 | | 0.55 | | 2.72 |
| Family history | | |  | |  |  | |  | |  | |  | |  | |  | |  | |  | |  | |  |
| Psychiatric disorders | | |  | |  |  | |  | |  | |  | |  | |  | |  | | 1.20 | | 0.71 | | 2.02 |
| Substance use disorders | | |  | |  |  | |  | |  | |  | |  | |  | |  | | 1.04 | | 0.58 | | 1.85 |
| Model comparison | *F* | *df1* | | *df2* | | | *p* | |  | |  | |  | |  | |  | |  | |  | |  | |
| Model 1 v 0 | **16.35** | **4** | | **1024** | | | **< .001** | |  | |  | |  | |  | |  | |  | |  | |  | |
| Model 2 v 1 | **5.72** | **3** | | **1021** | | | **< .001** | |  | |  | |  | |  | |  | |  | |  | |  | |
| Model 3 v 2 | **4.55** | **4** | | **1017** | | | **.001** | |  | |  | |  | |  | |  | |  | |  | |  | |
| Model 4 v 3 | 0.92 | 10 | | 1007 | | | .516 | |  | |  | |  | |  | |  | |  | |  | |  | |

**Table S6**. Unstandardized regression coefficients from the logistics regressions which predict SUD diagnoses at the 12-month study visits based on latent profiles from the baseline study visit: 5-class solution

|  | | | Model 1 | | | | | Model 2 | | | | | | Model 3 | | | | | | Model 4 | | | | |
| --- | --- | --- | --- | --- | --- | --- | --- | --- | --- | --- | --- | --- | --- | --- | --- | --- | --- | --- | --- | --- | --- | --- | --- | --- |
|  | | | *OR* | | *95% CI LL* | *95% CI UL* | | *OR* | | *95% CI LL* | | *95% CI UL* | | *OR* | | *95% CI LL* | | *95% CI UL* | | *OR* | | *95% CI LL* | | *95% CI UL* |
| Latent profiles | | |  | |  |  | |  | |  | |  | |  | |  | |  | |  | |  | |  |
| Low-Symptomatology | | | Ref | | Ref | Ref | | Ref | | Ref | | Ref | | Ref | | Ref | | Ref | | Ref | | Ref | | Ref |
| Intermediate Exposure-High PTSD | | | 1.06 | | 0.41 | 2.75 | | 0.46 | | 0.13 | | 1.68 | | 0.42 | | 0.11 | | 1.58 | | 0.4 | | 0.10 | | 1.56 |
| Intermediate Exposure-Moderate PTSD | | | 1.43 | | 0.74 | 2.77 | | 0.95 | | 0.45 | | 2.02 | | 0.85 | | 0.38 | | 1.89 | | 0.78 | | 0.34 | | 1.80 |
| Intermediate Exposure-High SU | | | **11.27** | | **6.00** | **21.15** | | **3.09** | | **1.41** | | **6.77** | | **3.03** | | **1.36** | | **6.76** | | **2.47** | | **1.06** | | **5.76** |
| High-Symptomatology | | | **13.01** | | **6.38** | **26.51** | | 1.77 | | 0.56 | | 5.67 | | 1.65 | | 0.49 | | 5.51 | | 1.35 | | 0.39 | | 4.72 |
| Baseline PTSD diagnosis | | |  | |  |  | | 0.95 | | 0.46 | | 2.00 | | 0.92 | | 0.43 | | 1.94 | | 0.89 | | 0.40 | | 1.98 |
| Baseline SUD diagnosis | | |  | |  |  | | **8.94** | | **4.78** | | **16.71** | | **8.64** | | **4.55** | | **16.40** | | **9.72** | | **4.91** | | **19.25** |
| Criterion A lifetime trauma | | |  | |  |  | | **1.14** | | **1.04** | | **1.26** | | **1.12** | | **1.02** | | **1.24** | | **1.10** | | **1.00** | | **1.22** |
| Criterion A trauma exposure between study visits | | |  | |  |  | |  | |  | |  | | **1.14** | | **1.02** | | **1.28** | | **1.14** | | **1.01** | | **1.29** |
| Psychotropic medication between study visits | | |  | |  |  | |  | |  | |  | | 0.88 | | 0.49 | | 1.57 | | 0.94 | | 0.51 | | 1.73 |
| Psychotherapy between study visits | | |  | |  |  | |  | |  | |  | | 1.36 | | 0.64 | | 2.86 | | 1.41 | | 0.63 | | 3.13 |
| Psychiatric hospitalization between study visits | | |  | |  |  | |  | |  | |  | | 0.87 | | 0.46 | | 1.67 | | 0.94 | | 0.48 | | 1.83 |
| Age | | |  | |  |  | |  | |  | |  | |  | |  | |  | | **1.20** | | **1.04** | | **1.39** |
| Male | | |  | |  |  | |  | |  | |  | |  | |  | |  | | 1.08 | | 0.59 | | 1.96 |
| Race | | |  | |  |  | |  | |  | |  | |  | |  | |  | |  | |  | |  |
| White | | |  | |  |  | |  | |  | |  | |  | |  | |  | | Ref | | Ref | | Ref |
| Black | | |  | |  |  | |  | |  | |  | |  | |  | |  | | 1.09 | | 0.46 | | 2.60 |
| Other minority | | |  | |  |  | |  | |  | |  | |  | |  | |  | | 1.24 | | 0.48 | | 3.20 |
| Multiracial | | |  | |  |  | |  | |  | |  | |  | |  | |  | | 1.83 | | 0.82 | | 4.07 |
| Hispanic | | |  | |  |  | |  | |  | |  | |  | |  | |  | | 0.90 | | 0.49 | | 1.65 |
| Household income | | |  | |  |  | |  | |  | |  | |  | |  | |  | |  | |  | |  |
| < $25,000 | | |  | |  |  | |  | |  | |  | |  | |  | |  | | Ref | | Ref | | Ref |
| $25,000 - $99,999 | | |  | |  |  | |  | |  | |  | |  | |  | |  | | **2.22** | | **1.08** | | **4.56** |
| > $99,999 | | |  | |  |  | |  | |  | |  | |  | |  | |  | | 1.50 | | 0.63 | | 3.56 |
| Family history | | |  | |  |  | |  | |  | |  | |  | |  | |  | |  | |  | |  |
| Psychiatric disorders | | |  | |  |  | |  | |  | |  | |  | |  | |  | | 0.89 | | 0.48 | | 1.67 |
| Substance use disorders | | |  | |  |  | |  | |  | |  | |  | |  | |  | | 1.29 | | 0.65 | | 2.59 |
| Model comparison | *F* | *df1* | | *df2* | | | *p* | |  | |  | |  | |  | |  | |  | |  | |  | |
| Model 1 v 0 | **26.83** | **4** | | **1024** | | | **< .001** | |  | |  | |  | |  | |  | |  | |  | |  | |
| Model 2 v 1 | **17.65** | **3** | | **1021** | | | **< .001** | |  | |  | |  | |  | |  | |  | |  | |  | |
| Model 3 v 2 | 1.44 | 4 | | 1017 | | | .219 | |  | |  | |  | |  | |  | |  | |  | |  | |
| Model 4 v 3 | 1.26 | 10 | | 1007 | | | .248 | |  | |  | |  | |  | |  | |  | |  | |  | |

**Figure S4**. Probability of endorsing trauma items by latent profile membership


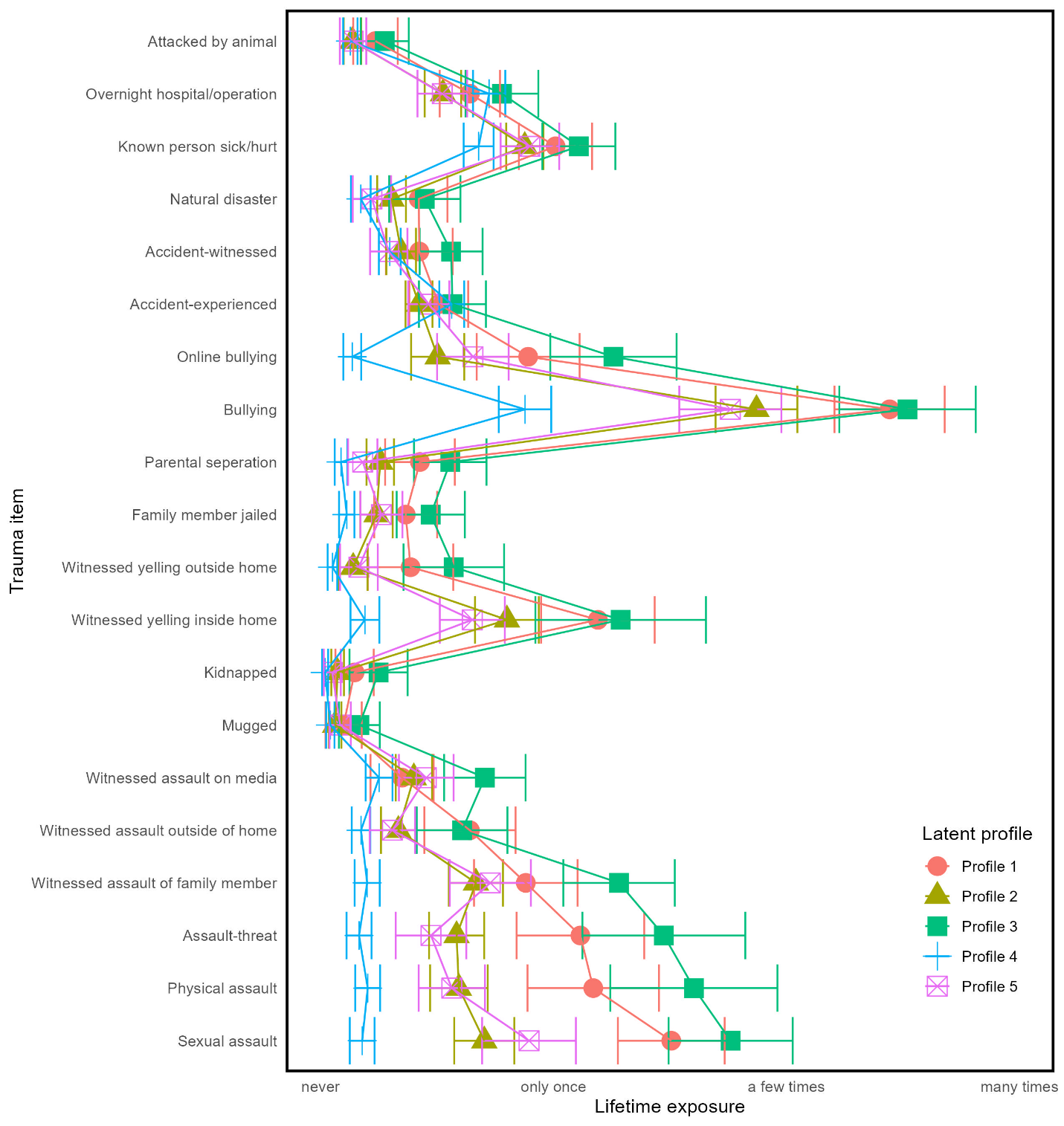


*Note*. The figure shows the posterior probability of endorsing each trauma item and the 95% confidence interval for each profile.

**Figure S5**. Probability of endorsing a posttraumatic stress disorder symptom by latent profile membership

**
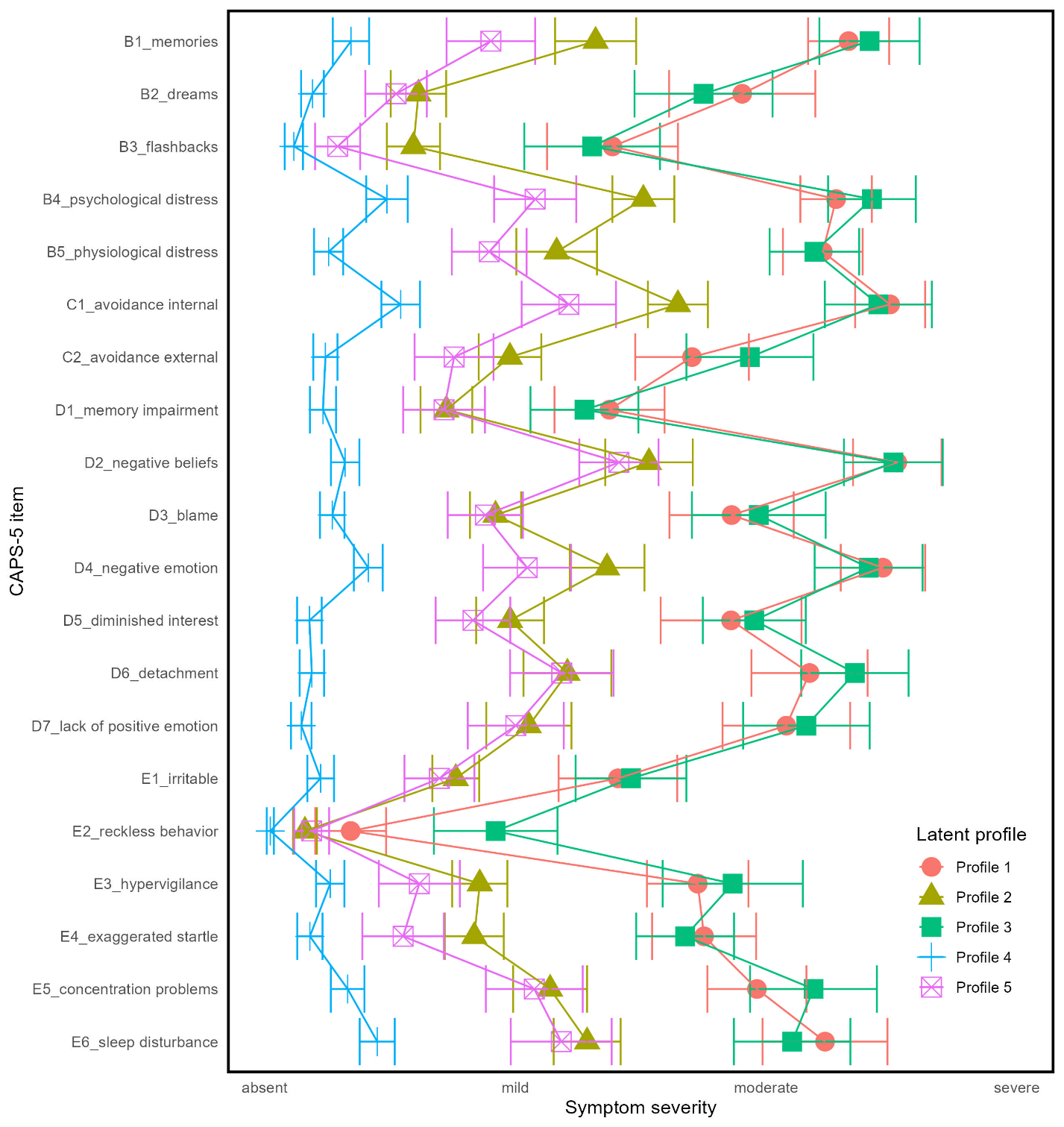
**

*Note*. The figure shows the posterior probability of endorsing each CAPS-CA-5 and the 95% confidence interval for each profile.

**Figure S6.** Probability of endorsing substance use items by latent profile membership


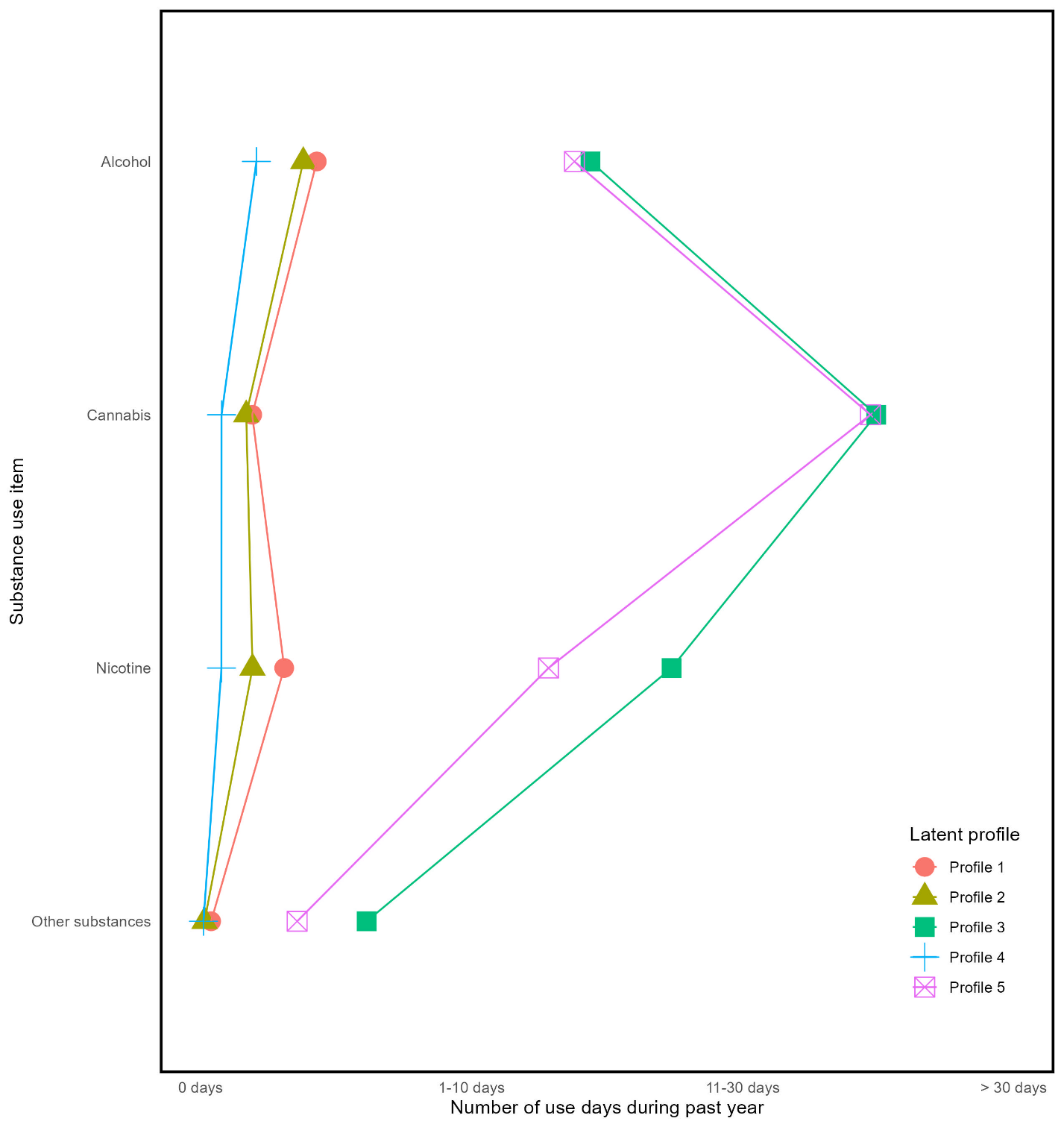


*Note*. The figure shows the posterior probability of endorsing each substance use item and the 95% confidence interval for each profile.
